# VCP promotes tTAF-target gene expression and spermatocyte differentiation by downregulating mono-ubiquitylated H2A

**DOI:** 10.1242/dev.201557

**Published:** 2023-07-19

**Authors:** Tyler J. Butsch, Olga Dubuisson, Alyssa E. Johnson, K. Adam Bohnert

**Affiliations:** Department of Biological Sciences, Louisiana State University, 202 Life Sciences Building, Baton Rouge, LA 70803, USA

**Keywords:** VCP, Meiosis, Spermatocyte, tTAF, Polycomb, Spermatogenesis, *Drosophila*

## Abstract

Valosin-containing protein (VCP) binds and extracts ubiquitylated cargo to regulate protein homeostasis. VCP has been studied primarily in aging and disease contexts, but it also affects germline development. However, the precise molecular functions of VCP in the germline, particularly in males, are poorly understood. Using the *Drosophila* male germline as a model system, we find that VCP translocates from the cytosol to the nucleus as germ cells transition into the meiotic spermatocyte stage. Importantly, nuclear translocation of VCP appears to be one crucial event stimulated by testis-specific TBP-associated factors (tTAFs) to drive spermatocyte differentiation. VCP promotes the expression of several tTAF-target genes, and *VCP* knockdown, like tTAF loss of function, causes cells to arrest in early meiotic stages. At a molecular level, VCP activity supports spermatocyte gene expression by downregulating a repressive histone modification, mono-ubiquitylated H2A (H2Aub), during meiosis. Remarkably, experimentally blocking H2Aub in *VCP-*RNAi testes is sufficient to overcome the meiotic-arrest phenotype and to promote development through the spermatocyte stage. Collectively, our data highlight VCP as a downstream effector of tTAFs that downregulates H2Aub to facilitate meiotic progression.

## Teaser

**Summary:** In the *Drosophila* male germline, VCP translocates from the cytosol to the nucleus during meiosis and directs downregulation of mono-ubiquitylated H2A to support spermatocyte gene expression and differentiation.

## INTRODUCTION

Valosin-containing protein (VCP; also known as TER94) is a broadly expressed protein that functions as a hexameric AAA+ ATPase to regulate protein homeostasis by binding ubiquitylated substrates and targeting them for degradation (Ye et al., 2017). Given its essential activities in protein homeostasis, VCP has been studied extensively in relation to aging and degenerative diseases (Darwich et al., 2020; Johnson et al., 2015; Kakizuka, 2008; Laço et al., 2012; Ritson et al., 2010; Scarian et al., 2022). However, VCP also acts in a variety of other biological contexts, including germ-cell development. In oocytes of the nematode *Caenorhabditis elegans*, CDC-48/VCP is particularly significant during meiotic divisions, when it regulates chromosome condensation and segregation (Mouysset et al., 2008; Sasagawa et al., 2012, 2009, 2007). Although there are indications that VCP may likewise perform regulatory roles during sperm development (Wu et al., 2021), it is unclear at what stage VCP function may be most crucial. Stage-specific transcriptomic data from *Drosophila* testes suggest that *VCP* is strongly expressed in meiotic-stage spermatocytes (Shi et al., 2020). Although this hints at a potentially important role for VCP at the spermatocyte stage, the molecular functions of VCP in meiotic spermatocytes are unknown.

**Fig. 1.**
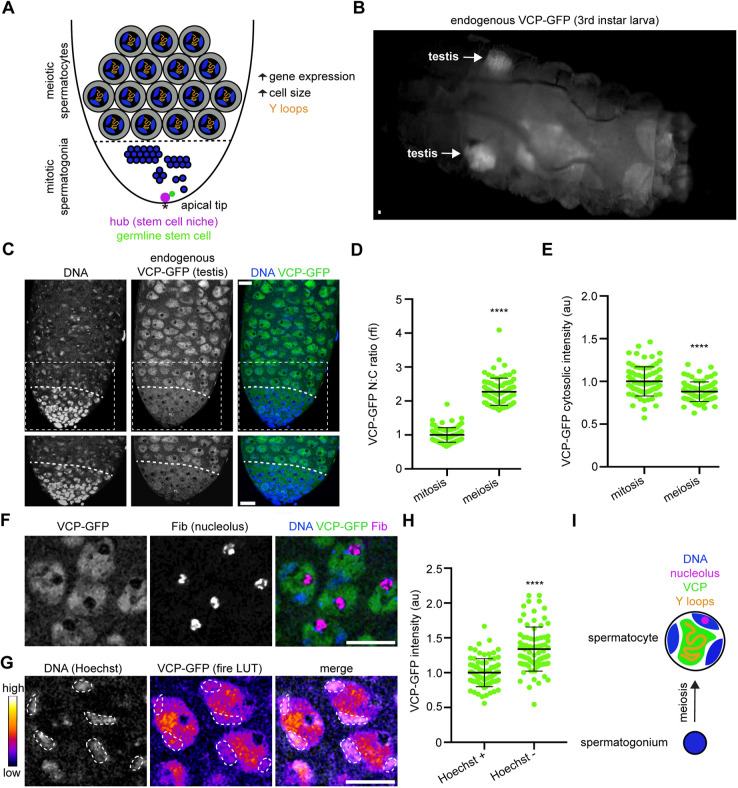
**VCP expression is high in the *Drosophila* testis, particularly in spermatocyte nuclei.** (A) Schematic of *Drosophila* spermatogenesis. The asterisk indicates the apical tip of the testis, where germline stem cells (green) and their niche (magenta) are located. Mitotic spermatogonia are represented by solid blue circles, which correspond to chromatin. The dashed line indicates the mitotic-meiotic transition, when mitotic spermatogonia differentiate into meiotic spermatocytes (tri-lobed structures corresponding to paired bivalents in the nucleus). Spermatocytes exhibit significant cell growth and upregulated gene expression (right). (B) Image of a VCP-GFP third instar larva. The arrows indicate larval testes. (C) Images of Hoechst (DNA) and VCP-GFP in an adult testis. Images below are magnified images of the outlined region in the top images. The dashed lines indicate the mitotic-meiotic transition. (D) Quantification of the nuclear-cytosolic ratio (N:C) of VCP-GFP intensity in mitotic spermatogonia (*n=*80 cells from 16 testes) and meiotic spermatocytes (*n=*80 cells from 16 testes). Mean±s.d. ****P<0.0001. Wilcoxon matched pairs signed rank test. (E) Quantification of cytosolic VCP-GFP intensity in mitotic spermatogonia (*n=*80 cells from 16 testes) and meiotic spermatocytes (*n=*80 cells from 16 testes). Mean±s.d. ****P<0.0001. Paired *t*-test. (F) Images of Hoechst (DNA), VCP-GFP and Fibrillarin immunostaining (Fib, nucleolus) in spermatocytes. (G) Images of Hoechst (DNA) and a heatmap of VCP-GFP signal in spermatocytes. Autosomes are outlined. (H) Quantification of VCP-GFP intensity at autosomes (outlined in G, Hoechst^+^) and in the interior of the nucleus (Hoechst^−^). Mean±s.d. ****P<0.0001. Welch's *t*-test. (I) Schematic showing that VCP localizes to the interior of the nucleus, near actively transcribed chromatin and Y-loops, after it enters the nucleus at the spermatogonia to spermatocyte transition. Scale bars: 20 µm. au, arbitrary units; rfi, relative fluorescence intensity. See also Fig. S1.

*Drosophila* spermatogenesis presents an ideal model in which to investigate conserved mechanisms regulating male germ-cell development, as the steps involved in making a viable sperm cell are similar in flies and mammals (Griswold, 2016; Hennig, 1992). At the apical tip of the *Drosophila* testis, germline stem cells divide, producing a daughter cell that undergoes four rounds of mitotic division to yield 16 spermatogonia (Fig. 1A). Subsequently, spermatogonia differentiate into spermatocytes, which enter meiotic prophase (Fig. 1A). Meiotic spermatocytes can be readily identified based on nuclear morphology; at this stage, nuclei are significantly larger than in spermatogonia, homologous chromosomes occupy distinct territories in the nucleus, and actively transcribed regions of the Y chromosome, termed Y-loops, extend into the nuclear interior (Fig. 1A) (Bonaccorsi et al., 1988; Cenci et al., 1994; Fingerhut et al., 2019; Mahadevaraju et al., 2021). Notably, extensive transcriptional rewiring occurs at meiotic prophase during spermatogenesis in flies (Fuller, 1998; Li et al., 2022; Mahadevaraju et al., 2021; White-Cooper, 2010; Witt et al., 2021), as in humans (Jan et al., 2017). These changes to transcription promote meiotic progression and are also needed to support later stages in germ-cell development (Lin et al., 1996; White-Cooper, 2010; White-Cooper et al., 1998). In *Drosophila*, testis-specific TBP-associated factors (tTAFs) are required to stimulate the spermatocyte gene expression program (Chen et al., 2005, 2011; Fuller, 1998; Hiller et al., 2004; White-Cooper et al., 1998). When tTAFs are non-functional, germ cells arrest as spermatocytes, causing infertility in male flies (Lin et al., 1996). Notably, a common form of human male infertility, non-obstructive azoospermia with spermatocyte arrest, exhibits similar cellular and developmental defects (Meyer et al., 1992; Soderström and Suominen, 1980) as tTAF mutants (Lin et al., 1996). Identifying factors that support robust spermatocyte gene expression, perhaps in cooperation with tTAFs, could reveal fundamental controls relevant to the preservation of male fertility.

Molecularly, tTAFs have been postulated to drive spermatocyte gene expression by antagonizing the activity of a transcriptional repressor, Polycomb (Pc) (Chen et al., 2005, 2011). Pc is a core component of Polycomb repressive complex I (PRC1), which inhibits the expression of target genes, including meiosis-related genes (Endoh et al., 2017; Zdzieblo et al., 2014), by catalyzing H2A mono-ubiquitylation (H2Aub) at repressed gene loci (Blackledge et al., 2020; de Napoles et al., 2004; Endoh et al., 2008; Wang et al., 2004). Interestingly, downregulation of PRC1 activity is a requisite step for meiotic entry and cellular differentiation in several developmental contexts (Tang et al., 2016; Yao et al., 2018; Zdzieblo et al., 2014). Despite these general themes, how downregulation of PRC1 activity is achieved at the molecular level during male germ-cell development remains incompletely understood, especially in relation to upstream signals from tTAFs. In principle, this may involve activation of factors that help to reverse repressive H2Aub epigenetic marks. However, the spatiotemporal regulation of H2Aub in the testis is unclear, as is the identity of enzymes that would facilitate its possible downregulation at meiosis.

In this study, we report that VCP operates downstream of tTAFs to downregulate H2Aub, thereby permitting spermatocyte gene expression and development in the *Drosophila* testis. Interestingly, we find that VCP is cytosolic in mitotic spermatogonia, but shuttles into the nucleus in meiotic spermatocytes. Nuclear translocation of VCP is dependent on tTAFs, and, like tTAF mutants, *VCP*-RNAi testes do not contain germ cells that develop beyond the spermatocyte stage. In line with these findings, we demonstrate that VCP is required to activate the expression of a subset of tTAF-regulated genes, and we observe that VCP translocation into the nucleus at meiotic entry coincides with a VCP-dependent downregulation of H2Aub. Remarkably, inhibiting PRC1 ubiquitin ligase activity is sufficient to advance sperm development past meiosis in the absence of VCP, suggesting that H2Aub downregulation is an important function of VCP in spermatocyte differentiation. Overall, our findings identify VCP as an essential regulator of both spermatocyte gene expression and meiotic progression.

## RESULTS

### VCP is highly expressed in the *Drosophila* testis and enters the nucleus as germ cells progress into meiotic prophase

We recently generated a collection of fly strains to assess VCP function in multisystem proteinopathy (MSP-1) (Wall et al., 2021). As part of these studies, we used CRISPR to knock-in a GFP tag at the endogenous *VCP* locus. While working with this strain, we noticed that VCP-GFP signal was exceptionally bright in third instar larval testes (Fig. 1B), which contain stem cells, spermatogonia and spermatocytes, but not later germ-cell stages (Gärtner et al., 2014). Interestingly, previous RNA-sequencing analyses have likewise suggested that VCP is highly expressed in the fly testis (Brown et al., 2014; Leader et al., 2018; Shi et al., 2020). Thus, we sought to analyze VCP localization and regulatory dynamics during *Drosophila* spermatogenesis in more detail.

We imaged adult VCP-GFP testes such that we could observe endogenous VCP expression patterns in a fully developed male germline. Although the protein was clearly expressed at multiple early stages in male germ-cell development, its localization varied depending on the developmental stage; VCP-GFP was primarily cytosolic in mitotic spermatogonia, but it translocated into the nucleus as cells developed into meiotic spermatocytes (Fig. 1C). Consequently, spermatocytes showed a higher nuclear-to-cytosolic VCP-GFP ratio compared with spermatogonia (Fig. 1D), and cytosolic VCP-GFP fluorescence was higher in spermatogonia than in spermatocytes (Fig. 1E). We confirmed that VCP enters the nucleus as germ cells exit mitosis and enter meiosis by imaging VCP-GFP in testes with germline-specific knockdown of *bag of marbles* (*bam*), which is required for mitotic spermatogonia to progress into the meiotic spermatocyte stage (McKearin and Spradling, 1990). Throughout *bam*-RNAi testes, VCP-GFP signal was restricted to the cytosol and was undetectable in nuclei (Fig. S1), indicating that the nuclear translocation event occurs after the spermatogonia stage. Interestingly, we found that VCP is excluded from the nucleolus (Fig. 1F) and that VCP-GFP signal is enriched in the interior of spermatocyte nuclei, away from Hoechst-labeled DNA (Fig. 1G-I). This subnuclear localization pattern is intriguing, given that open chromatin threads such as Y-loops also extend into the interior of the nucleus at the spermatocyte stage (Fig. 1I) (Bonaccorsi et al., 1988; Cenci et al., 1994; Fingerhut et al., 2019; Mahadevaraju et al., 2021), hinting at a possible role for VCP in transcription and gene expression. Overall, these data indicate that nuclear translocation of VCP is entrained to the spermatogonia to spermatocyte transition (Fig. 1I) and suggest a particularly important function for VCP in spermatocyte nuclei.

### Germline-specific knockdown of *VCP* arrests cells early in meiotic prophase and affects nuclear size

**Fig. 2.**
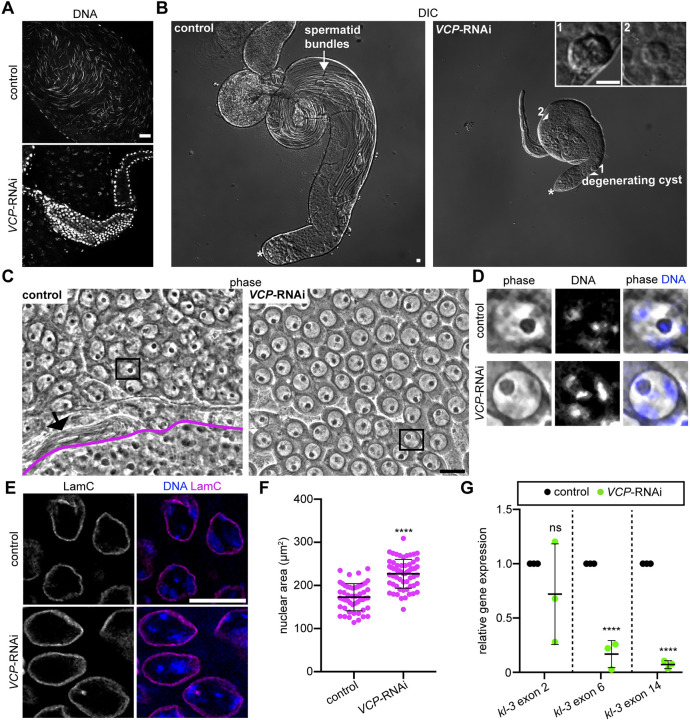
**VCP is required for male fertility, meiotic progression and nuclear regulation.** (A) Images of DNA (Hoechst) in control (BamGal4/+) and *VCP*-RNAi (BamGal4>*VCP*-RNAi) seminal vesicles. Mature sperm nuclei are needle-shaped (control), whereas only epithelial cells of the seminal vesicle are labeled in *VCP*-RNAi testes. (B) Low-magnification DIC images showing whole control and *VCP*-RNAi testes. The arrow indicates elongated spermatid bundles in control testes. The arrowheads indicate degenerating germ-cell cysts in *VCP*-RNAi testes, which are also magnified in insets. Numbers next to each arrowhead correspond to each inset in the top right corner. The asterisks indicate the apical tip of each testis. (C) Phase-contrast images of control (BamGal4/+) and *VCP*-RNAi (BamGal4>*VCP*-RNAi) squashed preparations. The black arrow indicates elongated spermatid tails in the control panel. Round spermatids in the onion stage are below the magenta line in the control panel. All other cells are spermatocytes. (D) Phase-contrast images of Hoechst (DNA)-labeled control and *VCP*-RNAi spermatocytes outlined in C. (E) Images of Hoechst (DNA) and Lamin C immunostaining (LamC) in spermatocytes of control and *VCP*-RNAi testes. (F) Quantification of nuclear area in control (*n=*50 spermatocytes from 10 testes) and *VCP*-RNAi (*n=*60 spermatocytes from 12 testes) testes. Mean±s.d. ****P<0.0001. Unpaired *t*-test. (G) Relative gene expression for the indicated *kl-3* exons. Gene expression was normalized to *Actin 5C* and then further normalized to *Cyclin A* for each genotype (*n=*3 replicates). Mean±s.d. ns, not significant (*P*>0.05); ****P<0.0001. Unpaired *t*-test. Scale bars: 20 µm. See also Fig. S2.

Given the redistribution of VCP into the nucleus at meiotic prophase, we hypothesized that VCP may play an important role in post-mitotic stages of sperm development. Because null *VCP* mutants are lethal (León and McKearin, 1999), we used a germline-specific Gal4 driver, BamGal4, to knock down *VCP* in the germline. Importantly, this approach successfully knocked down *VCP* in spermatocytes, but *VCP* expression in spermatogonia and cyst cells remained unaffected (Fig. S2A); thus, this presented a suitable approach to evaluate the developmental competency of germ cells upon knockdown of *VCP* in spermatocytes. As an initial assessment of whether mature sperm could even be produced in the absence of VCP, we first analyzed seminal vesicles. Not only were *VCP*-RNAi seminal vesicles noticeably shrunken, they were also devoid of mature sperm, as indicated by an absence of Hoechst-labeled, needle-shaped sperm nuclei (Fig. 2A). Using differential interference contrast (DIC) imaging, we confirmed that *VCP*-RNAi testes, which at a larger tissue level were also atypically small, lacked elongated spermatid bundles (Fig. 2B). Instead, the smaller *VCP-*RNAi testes appeared to show germ cells arrested at an earlier stage of development alongside some degenerating, post-mitotic germ-cell cysts (Fig. 2B, arrowheads). Consistent with a defect in mature sperm production, *VCP*-RNAi males were completely infertile (Fig. S2B).

To gain information regarding the precise developmental stage at which germ-cell development arrests in the absence of VCP, we stained DNA with Hoechst and performed high-magnification, phase-contrast microscopy on testis squashes. In control squashed preparations, we observed spermatocytes, round spermatids and elongated spermatids (Fig. 2C, left). However, elongated spermatids and round spermatids were absent from *VCP*-RNAi squashed preparations (Fig. 2C, right). Instead, we observed an accumulation of spermatocytes with well-formed nucleoli and condensed chromosomes that occupied distinct territories in the nucleus, similar to stage-matched control spermatocytes (Fig. 2C,D). Interestingly, *VCP*-RNAi spermatocyte nuclei appeared to be larger than control spermatocyte nuclei (Fig. 2D). We investigated this observation further by labeling Lamin C and measuring nuclear area in control and *VCP*-RNAi spermatocytes. Consistent with our initial observation, nuclear area was significantly increased in *VCP*-RNAi spermatocytes (Fig. 2E,F), suggesting that VCP may extract cargo from spermatocyte nuclei, as it does in *Drosophila* photoreceptors (Chang et al., 2021). Additionally, we noted that Hoechst staining appeared brighter in *VCP*-RNAi spermatocytes compared with controls (Fig. 2D), possibly due to hyper-condensation of chromatin. These data indicate that VCP is required for progression beyond the spermatocyte stage, the stage at which VCP enters the nucleus, and suggest that VCP may be important for nuclear and/or chromatin regulation.

The spermatocyte stage lasts approximately 80-90 h (Chandley and Bateman, 1962) and can be classified into sub-stages based on changes to chromatin morphology (Cenci et al., 1994). The chromatin of *VCP*-RNAi spermatocytes most closely resembled that of S3/S4-stage control spermatocytes (Fig. 2D), which are relatively early in spermatocyte development (Cenci et al., 1994). However, because VCP appeared to possibly regulate chromatin condensation and dynamics (Fig. 2D), we sought to confirm that germ cells arrested at an early-spermatocyte stage in *VCP*-RNAi testes by using a measure other than chromatin appearance. For this purpose, we analyzed the expression of gene regions of a Y-chromosome gene, *kl-3*. Notably, *kl-3* is transcribed in a stepwise fashion, with 5′ gene regions being transcribed early in spermatocyte development and gene regions distal to the 5′ end being transcribed later in spermatocyte development (Fingerhut et al., 2019). Interestingly, whereas expression of *kl-3* exon 2 was not robustly affected by *VCP* knockdown (Fig. 2G), expression of the more distal *kl-3* exons 6 and 14, which are expressed at later spermatocyte stages (Fingerhut et al., 2019), was strongly reduced in *VCP*-RNAi testes (Fig. 2G). These data suggest that knockdown of *VCP* causes cells to arrest early in the spermatocyte stage, at a time point shortly after when we observe VCP entering the nucleus (Fig. 1C).

### VCP promotes spermatocyte gene expression downstream of tTAFs

**Fig. 3.**
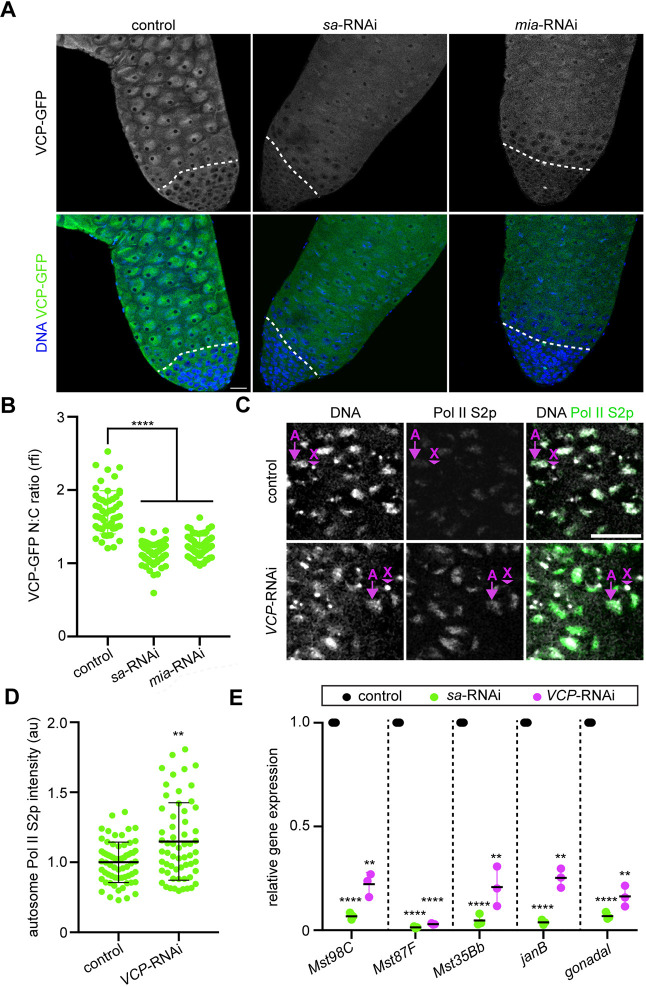
**VCP enters spermatocyte nuclei downstream of tTAFs and drives spermatocyte gene expression.** (A) Images of VCP-GFP and DNA (Hoechst) in control (BamGal4/+), *sa*-RNAi (BamGal4>*sa*-RNAi) and *mia*-RNAi (BamGal4>*mia*-RNAi) testes. The dashed lines indicate the mitotic-meiotic transition. Scale bar: 20 µm. (B) Quantification of the nuclear-cytosolic ratio (N:C) of VCP-GFP intensity in control (*n=*55 spermatocytes from 11 testes), *sa*-RNAi (*n=*90 spermatocytes from 18 testes) and *mia*-RNAi (*n=*65 spermatocytes from 13 testes) testes. Mean±s.d. ****P<0.0001. Ordinary one-way ANOVA with Dunnett's multiple comparisons test. (C) Images of Hoechst (DNA) and Pol II S2p immunostaining in control (BamGal4/+) and *VCP*-RNAi (BamGal4>*VCP*-RNAi) spermatocytes. The arrows indicate autosomes (labeled A), and the arrowheads indicate the X-chromosome (labeled X), which can be readily identified as a brightly stained dot that is near nucleoli. (D) Quantification of autosome Pol II S2p fluorescence intensity in control (*n=*65 spermatocytes from 13 testes) and *VCP*-RNAi (*n=*60 spermatocytes from 12 testes) spermatocytes. Mean±s.d. **P<0.01. Mann–Whitney U-test. (E) Relative gene expression for the indicated tTAF-target genes. Gene expression was normalized to *Actin 5C* and then further normalized to *Cyclin A* for each genotype (*n=*3 replicates). Note that statistical comparisons were made between control and each RNAi line (control versus RNAi). Mean±s.d. **P<0.01; ****P<0.0001. Two-way ANOVA. au, arbitrary units; rfi, relative fluorescence intensity. See also Fig. S3.

Similar to VCP, tTAFs are required for progression beyond early spermatocyte stages (Lin et al., 1996). Because loss of VCP and tTAF function each cause a common developmental arrest at the spermatocyte stage, we hypothesized that VCP may function in the same pathway as tTAFs. Intriguingly, co-imaging of a GFP-tagged tTAF protein, Spermatocyte arrest (Sa-GFP), with antibody-labeled VCP indicated that VCP enters spermatocyte nuclei shortly after Sa is expressed in spermatocytes (Fig. S3A). Based on this timing, we were curious about whether tTAFs promote the nuclear translocation of VCP in spermatocytes. Indeed, knockdown of *sa* and another tTAF, *meiosis I arrest* (*mia*), impeded nuclear translocation of VCP in spermatocytes (Fig. 3A,B). However, knockdown of *VCP* did not reciprocally affect Sa expression or localization (Fig. S3B), indicative of a unidirectional pathway. Thus, we conclude that VCP depends upon tTAFs to enter spermatocyte nuclei.

A major function of tTAFs is to activate the spermatocyte gene expression program (Chen et al., 2005, 2011; White-Cooper et al., 1998). Given that VCP enters the nucleus downstream of tTAFs, we hypothesized that VCP regulates the expression of spermatocyte-specific genes as part of this program. To test this hypothesis, we first examined the localization and intensity of phospho-serine 2 RNA polymerase II (Pol II S2p), which is a marker of transcriptional elongation (Mahadevaraju et al., 2021; Phatnani and Greenleaf, 2006). In both control and *VCP*-RNAi spermatocytes, Pol II S2p was undetectable at the X-chromosome (Fig. 3A), suggesting that the X-chromosome is properly inactivated under each condition (Mahadevaraju et al., 2021; Witt et al., 2021). Unlike the X-chromosome, autosomes showed clear Pol II S2p signal in both control and *VCP*-RNAi spermatocytes (Fig. 3C). However, Pol II S2p fluorescence intensity was significantly brighter in *VCP*-RNAi spermatocytes (Fig. 3C,D), consistent with a change in transcription.

Because VCP functions in the same pathway as tTAFs and transcription is downregulated in tTAF mutants (Chen et al., 2005, 2011; Fuller, 1998), we hypothesized that increased Pol II S2p in spermatocytes of *VCP-*RNAi testes indicates stalled transcription, rather than enhanced transcription. We tested this hypothesis by performing RT-qPCR to measure quantitatively the expression of a subset of tTAF-regulated genes [*Mst98C*, *Mst87F*, *Mst35Bb* (*ProtB*), *janB*, *gonadal*] (White-Cooper et al., 1998) in control and *VCP*-RNAi testes. As an additional control to verify tTAF dependence, we performed similar analyses on *sa*-RNAi testes. To rule out possible artifacts caused by developmental arrest at early spermatocyte stages in *VCP­*-RNAi and *sa*-RNAi testes, we normalized tTAF-target gene expression to that of *Cyclin A*, which is uniformly expressed throughout spermatocyte development and is not regulated by tTAFs (Chen et al., 2011; White-Cooper et al., 1998); this allowed us to compare directly differences in gene expression specifically in relation to the gene knockdown of interest. As in *sa-*RNAi testes (Fig. 3E), we detected a sharp decrease in each of the five tested tTAF-regulated genes in *VCP*-RNAi testes (Fig. 3E). Thus, we conclude that VCP drives the expression of several tTAF-regulated spermatocyte genes, which could potentially explain the meiotic-arrest phenotype observed upon loss of VCP function.

### VCP and tTAFs stimulate H2Aub downregulation in spermatocytes

**Fig. 4.**
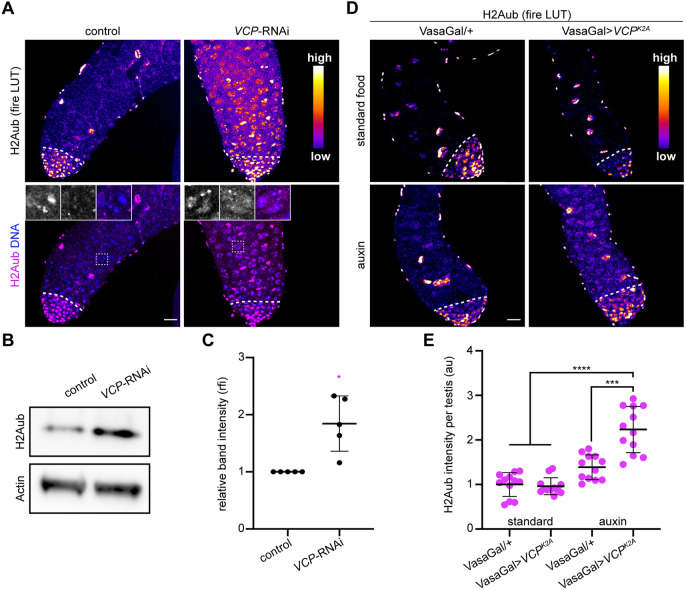
**VCP negatively regulates H2A mono-ubiquitylation.** (A) Top: Heatmap images of H2Aub in control (BamGal/+) and *VCP*-RNAi (BamGal>*VCP*-RNAi) testes. Bottom: Fluorescence images of H2Aub and Hoechst (DNA) in control (BamGal/+) and *VCP*-RNAi (BamGal>*VCP*-RNAi) testes. The dashed lines indicate the mitotic-meiotic transition. Magnifications of the outlined regions are shown in insets (DNA, left; H2Aub, middle; merge, right). (B) Western blotting for H2Aub (top) and Actin (bottom, loading control) in control and *VCP*-RNAi testes. (C) Quantification of H2Aub band intensity normalized to Actin band intensity (*n=*5 replicates). Mean±s.d. *P<0.05. Unpaired *t*-test. (D) Heatmap images of H2Aub in VasaGal4/+ and VasaGal4>*VCP^K2A^* testes from flies that were fed standard food or auxin for 10 days. The dashed lines indicate the mitotic-meiotic transition. (E) Quantification of H2Aub intensity in spermatocytes of flies of the indicated genotypes fed the indicated types of food. Mean±s.d. ***P<0.001; ****P<0.0001. Brown–Forsythe and Welch ANOVA. Scale bars: 20 µm. au, arbitrary units; rfi, relative fluorescence intensity. See also Figs S4 and S5.

We next investigated how VCP supports gene expression downstream of tTAFs. Notably, tTAFs have been postulated to antagonize PRC1-mediated repression of meiotic gene expression in spermatocytes (Chen et al., 2005, 2011). Strikingly, we found that H2Aub, a repressive histone mark catalyzed by PRC1, decreased in wild-type testes specifically at the spermatocyte stage; H2Aub was bright in spermatogonia and somatic cyst cells throughout the testis, but H2Aub signal declined dramatically in spermatocytes (Fig. S4A,B). In multiple contexts, VCP binds and extracts ubiquitylated cargo (Ye et al., 2017). As the reduction in H2Aub during spermatogenesis coincided with nuclear entry of VCP, we tested whether H2Aub downregulation in spermatocytes depends upon VCP function. Indeed, high H2Aub signal was retained in spermatocytes and was uniformly distributed throughout the spermatocyte nucleoplasm when *VCP* was knocked down (Fig. 4A, insets), potentially owing to a failure to extract H2Aub from spermatocyte nuclei. Additionally, H2Aub was significantly more abundant in protein extracts from whole testes (Fig. 4B,C). Knockdown of *sa* likewise showed an increase in H2Aub in spermatocytes (Fig. S5A) and protein extracts from whole testes (Fig. S5B,C), as would be expected if VCP operated downstream of tTAFs to trigger the downregulation of H2Aub.

To corroborate that H2Aub downregulation during spermatogenesis was dependent on VCP activity, we overexpressed an allele of *VCP* that lacks ATPase activity (*VCP^K2A^*) (Chang et al., 2021). Because VCP functions as a hexamer (Wang et al., 2003), overexpression of *VCP^K2A^* causes dominant-negative effects by generating hexamers containing the ATPase-dead protein in complex with the wild-type protein, thus impeding VCP function. For this approach, we were unable to drive *VCP^K2A^* overexpression using BamGal4, as this caused lethality prior to adulthood. To overcome this experimental limitation, we utilized an auxin-inducible gene expression system (McClure et al., 2022) to restrict transgene expression to adults. This system utilizes a ubiquitously expressed Gal4 inhibitor, Gal80, fused to an auxin-inducible degron (AID). Upon auxin feeding, auxin binds Gal80 and causes its degradation, thus permitting Gal4 activity and transgene expression. For this experiment, we used the VasaGal4 driver, which is active in all germ cells (Demarco et al., 2014) and should, in principle, provide the strongest transgene expression. Consistent with our *VCP*-RNAi data, H2Aub signal was higher in spermatocytes of auxin-fed *VCP^K2A^* males relative to controls (Fig. 4D,E). Thus, we conclude that VCP ATPase activity is indeed required for H2Aub downregulation in spermatocytes.

### VCP supports H2A extraction and turnover

**Fig. 5.**
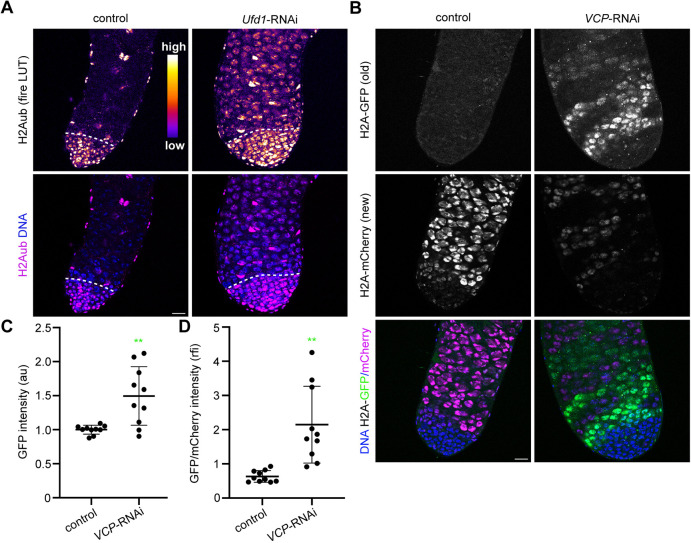
**VCP cooperates with the co-factor Ufd1 and promotes H2A turnover.** (A) Top: Heatmap images of H2Aub in control (BamGal/+) and *Ufd1*-RNAi (BamGal>*Ufd1*-RNAi) testes. Bottom: Fluorescence images of H2Aub and Hoechst (DNA) in control (BamGal/+) and *Ufd1*-RNAi (BamGal>*Ufd1*-RNAi) testes. The dashed lines indicate the mitotic-meiotic transition. (B) Images of Hoechst (DNA), H2A-GFP (old H2A) and H2A-mCherry (new H2A) in control (BamGal/+) and *VCP*-RNAi (BamGal>*VCP*-RNAi) testes. Testes were dissected and imaged 5 days after FLPase induction (H2A-GFP cassette removal). (C) Quantification of GFP intensity in spermatocytes of control (BamGal/+) and *VCP*-RNAi (BamGal>*VCP*-RNAi) testes. Mean±s.d. **P<0.01. Welch's *t*-test. (D) Quantification of the GFP/mCherry ratio in spermatocytes of control (BamGal/+) and *VCP*-RNAi (BamGal>*VCP*-RNAi) testes (*n*=10 testes for both genotypes in both graphs). Mean±s.d. **P<0.01. Welch's *t*-test. Scale bars: 20 µm. au, arbitrary units; rfi, relative fluorescence intensity. See also Fig. S6.

To gain insight into how VCP may downregulate H2Aub, we screened several known VCP co-factors (Fig. S6A) for gene knockdowns that increased H2Aub in spermatocytes. Among these candidates, we found that only knockdown of *Ufd1*, which encodes a ubiquitin-recognition factor that binds VCP and supports the sequestration and degradation of ubiquitylated proteins (Ramadan et al., 2007; Sasagawa et al., 2009; Shcherbik and Haines, 2007), blocked H2Aub downregulation in spermatocytes (Fig. 5A, Fig. S6A). We conclude that VCP may cooperate with Ufd1 to drive H2Aub downregulation.

Because VCP and Ufd1 commonly act together to drive the extraction and degradation of ubiquitylated substrates, we hypothesized that VCP and Ufd1 may stimulate H2Aub downregulation by promoting the turnover of H2A in spermatocytes. Notably, the nuclear-swelling phenotype observed in *VCP*-RNAi spermatocytes (Fig. 2E,F) suggests that VCP may indeed control the extraction and degradation of various nuclear proteins, such as H2A. To test this hypothesis, we used BamGal4 to express a transgenic H2A-turnover reporter construct (FRT-H2A-GFP-FRT-H2A-mCherry) (Kahney et al., 2021). This system can be used to measure the turnover of fluorescently tagged, transgenic H2A, but it does not provide information regarding endogenous H2A turnover. To activate this system, we used heat shock to transiently induce expression of a FLPase, which removes the H2A-GFP cassette and permits H2A-mCherry expression. Given time, older H2A will remain marked by GFP, whereas nascent H2A will be marked by mCherry. We first verified the functionality of this system by amplifying the H2A-turnover transgene in extracts from testes with or without the FLPase transgene. In both control and *VCP-*RNAi testes, we observed more robust amplification of the smaller FRT-H2A-mCherry DNA cassette, as opposed to the full FRT-H2A-GFP-FRT-H2A-mCherry transgene, after FLPase induction (Fig. S6B), confirming that our approach effectively triggered recombination.

We then utilized this system to examine H2A turnover *in vivo* by microscopy. In control testes 5 days after heat shock, GFP signal was barely detectable above background levels whereas mCherry signal was bright (Fig. 5B, Fig. S6C), consistent with high H2A turnover under normal conditions. We then investigated whether *VCP* depletion would cause a significant retention in GFP signal, as would be expected if VCP were required for H2A turnover. Indeed, 5 days after heat shock, GFP signal remained bright in many spermatocyte cysts of *VCP*-RNAi testes (Fig. 5B, Fig. S6C). Quantification of GFP intensity and the GFP/mCherry ratio in spermatocytes within fields of view indicated a significantly higher mean GFP intensity (Fig. 5C) and GFP/mCherry ratio (Fig. 5D) in *VCP*-RNAi testes compared with controls (Fig. 5D). Despite this general increase in GFP signal relative to mCherry signal in *VCP*-RNAi testes, we reproducibly observed inter-cyst variability in the degree to which H2A turnover was inhibited (Fig. 5B, Fig. S6C). Whereas some cysts showed bright GFP signal with almost no detectable mCherry signal, other cysts showed dimmer GFP signal and relatively stronger mCherry signal, which nonetheless remained dimmer than in controls (Fig. 5B). Although the reason for this inter-cyst variability is currently unclear, the evident retention of more GFP signal in *VCP*-RNAi testes compared with controls supports the more general conclusion that VCP activity enables robust H2A turnover in spermatocytes, potentially contributing to VCP-dependent H2Aub downregulation.

Ubiquitylated substrates extracted by VCP are often targeted to the proteasome for degradation (Ye et al., 2017). Therefore, we next examined whether proteasomal degradation was also involved in H2Aub downregulation. Notably, mutation in *Rpt2*, a gene that codes for a proteasome component, causes a meiotic arrest resembling *VCP*-RNAi and tTAF mutant testes (Lu et al., 2013). Whereas germline-specific knockdown of *Rpt2* in fact blocked downregulation of H2Aub in spermatocytes (Fig. S6D), VCP remained almost completely cytosolic in spermatocytes and failed to effectively translocate into the nucleus (Fig. S6E). Thus, although it remains possible that the proteasome may also act downstream of VCP to degrade extracted H2Aub, proteasome function is apparently important for VCP nuclear translocation, which precludes us from definitively testing whether H2Aub is degraded by the proteasome after extraction in this context.

### Genetic inhibition of H2Aub promotes spermatocyte gene expression in *VCP*-RNAi testes

**Fig. 6.**
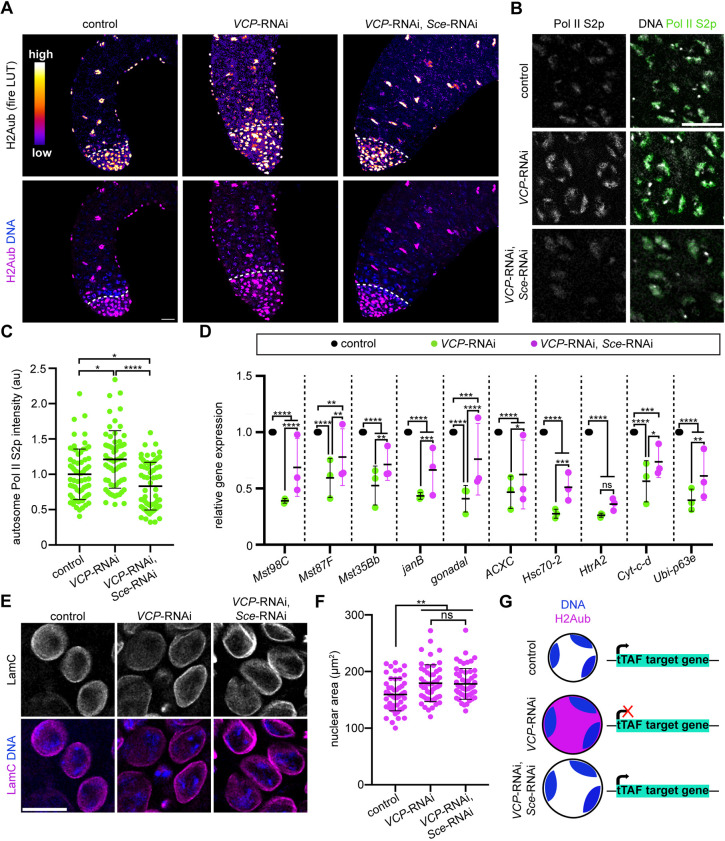
**Inhibition of H2A mono-ubiquitylation promotes gene expression in the absence of VCP.** (A) Top: Heatmap images of H2Aub in control (BamGal/+), *VCP*-RNAi (BamGal>*VCP*-RNAi), and DKD (BamGal>*VCP*-RNAi, *Sce*-RNAi) testes. Bottom panel: Fluorescence images of H2Aub and Hoechst (DNA) in control (BamGal/+), *VCP*-RNAi (BamGal>*VCP*-RNAi), and DKD (BamGal>*VCP*-RNAi, *Sce*-RNAi) testes. The dashed lines indicate the mitotic-meiotic transition. (B) Images of Pol II S2p in control, *VCP*-RNAi and DKD testes. (C) Quantification of autosome Pol II S2p fluorescence intensity in control (*n=*65 spermatocytes from 13 testes), *VCP*-RNAi (*n=*60 spermatocytes from 12 testes) and DKD (*n=*65 spermatocytes from 13 testes) spermatocytes. Mean±s.d. *P<0.05; ****P<0.0001. Kruskal–Wallis test. (D) Graph of relative gene expression for the indicated tTAF-target genes. Gene expression was normalized to *Actin 5C* and then further normalized to *Cyclin A* for each genotype (*n=*3 replicates). Mean±s.d. ns, not significant (*P*>0.05); *P<0.05; **P<0.01; ***P<0.001; ****P<0.0001. Two-way ANOVA. (E) Images of Hoechst (DNA) and Lamin C immunostaining (LamC) in spermatocytes of control, *VCP*-RNAi and DKD testes. (F) Quantification of nuclear area in control (*n=*50 spermatocytes from 10 testes), *VCP*-RNAi (*n=*50 spermatocytes from 10 testes) and DKD (*n=*55 spermatocytes from 11 testes) testes. Mean±s.d. **P<0.01. Unpaired *t*-test. (G) Schematic showing that VCP downregulates H2Aub to promote the expression of tTAF-target genes. au, arbitrary units. Scale bars: 20 µm.

H2Aub is a well-characterized repressive histone modification (Blackledge et al., 2020; de Napoles et al., 2004; Endoh et al., 2008; Wang et al., 2004). Given that VCP promotes spermatocyte gene expression and H2Aub downregulation, we hypothesized that inhibition of H2Aub in *VCP*-RNAi testes may at least partially rescue spermatocyte gene expression. To test this hypothesis, we used a BamGal4 driver to simultaneously knock down *VCP* and *sex combs extra* (*Sce*), the PRC1 E3 ubiquitin ligase that catalyzes H2Aub (de Napoles et al., 2004; Wang et al., 2004). As expected, H2Aub levels were dampened in spermatocytes of *VCP Sce* double-knockdown (DKD) testes, such that the pattern of H2Aub resembled that of control spermatocytes (Fig. 6A). Importantly, H2Aub did not appear to be affected in spermatogonia or cyst cells (Fig. 6A), indicating that *Sce* knockdown is restricted to spermatocytes. We next tested whether blocking H2Aub in the absence of VCP would restore normal Pol II S2p levels. Indeed, Pol II S2p intensity at autosomes was significantly decreased in DKD testes compared with *VCP*-RNAi testes (Fig. 6B,C), suggesting that normal transcription may be restored in DKD testes. Notably, each of the five tTAF-target genes we previously found to be downregulated in *VCP*-RNAi testes (Fig. 3C) increased in expression in DKD testes (Fig. 6D). To broaden our analysis, we analyzed five additional, putative tTAF targets (Jiang et al., 2018; White-Cooper et al., 1998) and confirmed that all but one (*HtrA2*) were indeed expressed significantly higher in DKD testes compared with *VCP*-RNAi testes (Fig. 6D). Despite the partial rescue of tTAF-target gene expression in DKD testes, blocking H2Aub in the absence of VCP did not suppress the nuclear swelling phenotype (Fig. 6E,F), suggesting that VCP-dependent regulation of tTAF-target gene expression may not be coupled to its regulation of nuclear size. Collectively, these data indicate that VCP downregulates H2Aub to drive the expression of several tTAF-target genes (Fig. 6G).

### Genetic blockade of H2Aub is sufficient to promote spermatocyte differentiation in the absence of VCP

**Fig. 7.**
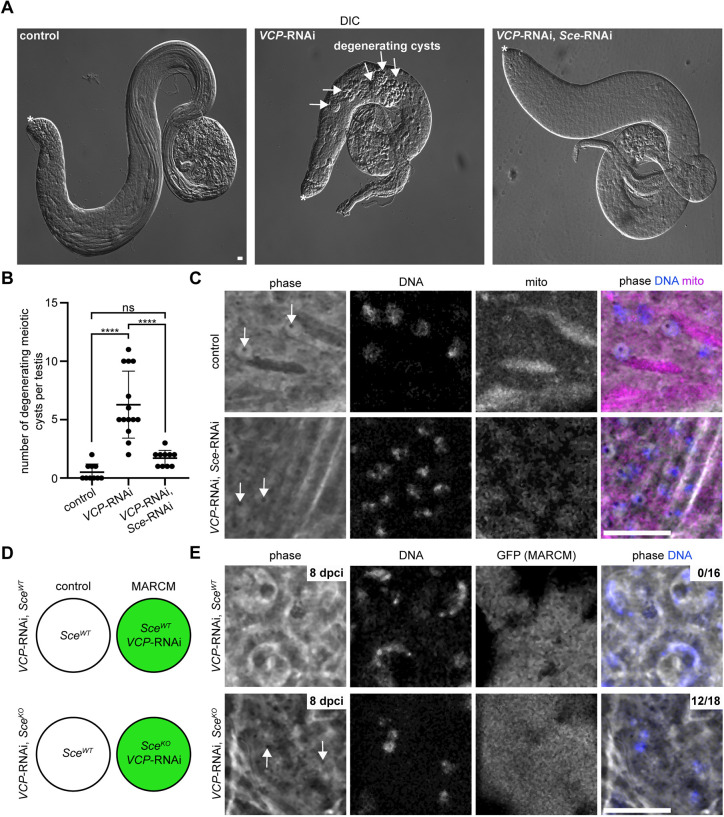
**Inhibition of H2A mono-ubiquitylation supports meiotic progression in the absence of VCP.** (A) Low-magnification DIC images of control (BamGal/+), *VCP*-RNAi (BamGal>*VCP*-RNAi) and DKD (BamGal>*VCP*-RNAi, *Sce*-RNAi) testes. The asterisks indicate the apical tip of each testis. The arrows indicate degenerating germ-cell cysts in *VCP*-RNAi testes. (B) Graph of the number of degenerating germ-cell cysts per testis in control (*n*=10 testes), *VCP*-RNAi (*n*=14 testes) and DKD (*n*=10 testes) testes. Mean±s.d. ns, not significant (*P*>0.05); ****P<0.0001. Ordinary one-way ANOVA. (C) Phase-contrast images of spermatids labeled with Hoechst (DNA) and MitoTracker (mito) in control and DKD testes. The arrow indicates the protein body, which is a marker of the elongating spermatid stage. (D) Schematic of the MARCM experimental setup. In *VCP*-RNAi, *Sce^WT^* testes, GFP-negative cells (top left) are homozygous wild type at the *Sce* locus and *VCP* is expressed normally. In these same testes, GFP-positive cells (top right) are wild type at the *Sce* locus and *VCP* is knocked down. In *VCP*-RNAi, *Sce^KO^* testes, GFP-negative cells (bottom left) are heterozygous for the *Sce^KO^* allele and *VCP* is expressed normally. In these same testes, GFP-positive cells (bottom right) are homozygous for the *Sce^KO^* allele and *VCP* is knocked down. (E) Phase-contrast images of GFP-positive, Hoechst (DNA)-labeled cells in *VCP*-RNAi, *Sce^WT^* (top) and *VCP*-RNAi, *Sce^KO^* (bottom) testes at 8 dpci. Cells in *VCP*-RNAi, *Sce^WT^* are spermatocytes; we never observed GFP-positive cells beyond the spermatocyte stage in these testes. The arrows indicate protein bodies. Numbers in the top right corner of the right-most images indicate the proportion of testes containing post-meiotic spermatids. Scale bars: 20 μm. See also Fig. S7.

Like *VCP* and *sa*, multiple de-repressed genes in DKD testes, including *Hsc70-2* and *Ubi-p63E*, are required for meiotic progression (Azuma et al., 2021; Lu et al., 2013). We therefore hypothesized that genetic inhibition of H2Aub may rescue some aspects of germ-cell development in *VCP*-RNAi testes. To test this hypothesis, we used microscopy to assess how far germ-cell development progressed in DKD testes. Although DKD testes still failed to produce elongated spermatid bundles or mature sperm (Fig. 7A), DKD testes were visibly larger than *VCP*-RNAi testes (Fig. 7A) and exhibited a significant reduction in degenerating post-mitotic germ-cell cysts, which were prevalent in *VCP*-RNAi testes (Fig. 7A,B).

We hypothesized that the increased testis size and suppression of cyst degeneration in DKD testes may be indicative of the presence of more-developed germ cells, such as post-meiotic spermatids. Post-meiotic spermatids are characterized by compact chromatin and the presence of a mitochondrial-based structure known as the nebenkern. As an initial assessment of potential spermatid development, we stained testes with Hoechst and a mitochondria-specific dye, MitoTracker. Remarkably, although VCP was non-functional, we observed post-meiotic germ cells with compact chromatin and a circular nebenkern structure adjacent to nuclei in DKD testes (Fig. S7A), indicating that DKD testes were indeed capable of producing post-meiotic spermatids. Importantly, double knockdown of *sa* and *Sce* did not similarly permit meiotic progression (Fig. S7B), suggesting that tTAFs likely perform some additional function independent of H2Aub downregulation that is needed to drive meiotic progression.

The presence of a circular nebenkern in DKD spermatids suggested that these cells may arrest at the round spermatid stage, an early sub-stage of spermatid development (Fabian and Brill, 2012). To probe the spermatid stage in more detail, we used phase-contrast microscopy. By this approach, we also detected spermatids with compact chromatin and non-elongated mitochondria in DKD testes (Fig. 7C); however, we noted that the nebenkern did not appear as a phase-dark structure in DKD spermatids (Fig. 7C), as it did in controls (Fig. 7C), hinting that VCP may also regulate mitochondrial morphogenesis in spermatids. Interestingly, although a circular nebenkern is characteristic of round spermatids, we also detected by phase-contrast microscopy a protein dot, which is more characteristic of elongating spermatids (Fabian and Brill, 2012). Thus, it is possible that the spermatids we detected in DKD testes may represent a hybrid and/or transitional state somewhere between the round and elongating spermatid stages.

As a complementary approach to assess the potential rescuing effects of *Sce* inhibition, we used mosaic analysis with a repressible cell marker (MARCM) to examine the effects of an *Sce^KO^* allele (Gutiérrez et al., 2012) in the germline. In this system (Lee and Luo, 1999), FLP-based recombination produces cells that are homozygous for the allele of interest (i.e. *Sce^KO^*) and do not express Gal80, a Gal4 inhibitor (Duffy, 2002); uninhibited Gal4, in turn, drives expression of a GFP marker that enables the identification of MARCM clones (Lee and Luo, 1999). In contrast, GFP-negative cells in the same tissue, which possess at least one functional copy of the gene of interest (i.e. *Sce*), express Gal80 and block Gal4 activity. With this set-up, we first ensured that GFP-positive *Sce^KO^* homozygotes could not mono-ubiquitylate H2A by imaging H2Aub in spermatogonia 2 days-post clone induction (dpci). As expected, GFP-positive *Sce^KO^* clones were negative for H2Aub, but neighboring GFP-negative clones were positive for H2Aub (Fig. S7C). Thus, this MARCM strategy provides a means to effectively block H2Aub cell-autonomously in developing germ cells.

Experimentally, the MARCM system can also be applied to drive RNAi-mediated knockdown of genes specifically in GFP-positive MARCM clones, but not in GFP-negative neighboring cells. In GFP-positive cells, Gal80 is absent, which permits Gal4 activity, and therefore expression of an RNAi transgene (i.e. *VCP*-RNAi). In GFP-negative cells, Gal80 is present, which inhibits Gal4 activity and RNAi transgene expression (Lee and Luo, 1999). We therefore used the MARCM system to generate germ-cell clones expressing *VCP*-RNAi that were also homozygous for an *Sce^KO^* allele, or, as a control, an *Sce^WT^* allele (Fig. 7D). We tested whether VCP was indeed knocked down in GFP-positive cells by labeling VCP with an antibody in MARCM testes. As expected, VCP was undetectable in GFP-positive germ-cell nuclei, although it remained detectable in neighboring GFP-negative germ cells (Fig. S7D). After confirming that this system functioned properly, we imaged testes at 7-9 dpci, when cells should be in post-meiotic stages if they developed normally (Chandley and Bateman, 1962). As a control, we imaged *VCP*-RNAi, *Sce^WT^* MARCM testes, which we found never contained post-meiotic GFP-positive cysts (0/16 testes); we observed only GFP-positive spermatocytes in these testes (Fig. 7E), consistent with an essential function of VCP at the spermatocyte stage. In contrast, GFP-positive spermatids were present in 12/18 *VCP*-RNAi, *Sce^KO^* MARCM testes (Fig. 7E). Similar to DKD testes, GFP-positive spermatids exhibited compact chromatin and a circular nebenkern in *VCP*-RNAi, *Sce^KO^* MARCM testes (Fig. S7E). Using phase-contrast imaging, we also observed a protein body at spermatid nuclei in *VCP*-RNAi, *Sce^KO^* MARCM testes (Fig. 7E), but were unable to detect the nebenkern as a phase-dark structure. Together with the DKD analysis, these data indicate that blocking H2Aub in the absence of VCP generates spermatids with features characteristic of both the round and elongating spermatid stages (i.e. circular nebenkern or protein body, respectively). Thus, although it appears that VCP must also execute additional functions to support germline development past the elongating spermatid stage, we conclude that VCP downregulates H2Aub in spermatocytes to promote spermatocyte differentiation.

## DISCUSSION

**Fig. 8.**
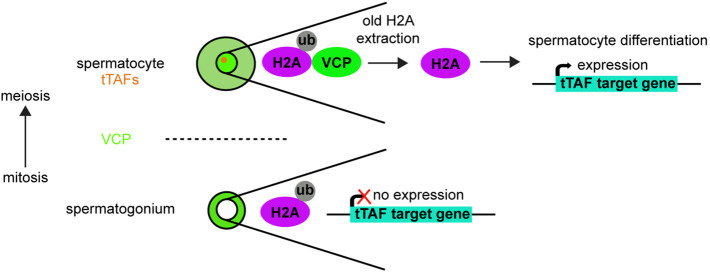
**Model for VCP in spermatocyte development.** In mitotic spermatogonia (below the dashed line), VCP (green) is cytosolic, H2Aub (H2A, magenta; ub, gray) is present, and tTAF-target genes (cyan) are not expressed. As spermatogonia differentiate into meiotic spermatocytes (above the dashed line), tTAFs (orange) are expressed and VCP enters spermatocyte nuclei. Nuclear entry of VCP triggers the extraction of old H2A and downregulation of H2Aub. The downregulation of H2Aub drives tTAF-target gene expression and spermatocyte differentiation.

Although previous studies have demonstrated that VCP influences germ-cell health and development (León and McKearin, 1999; Sasagawa et al., 2012, 2009, 2007; Wu et al., 2021), fundamental questions remain regarding how VCP functions and is regulated in this biological context, particularly in the male germline. In this study, we found that VCP translocates from the cytosol to the nucleus at the mitotic-meiotic transition and downregulates H2Aub to promote changes in gene expression needed for subsequent stages of spermatogenesis (Fig. 8). These findings present a new molecular framework for understanding developmental regulation of VCP and the mechanisms by which it ultimately controls spermatocyte development.

In the *Drosophila* testis, nuclear entry of VCP at the spermatocyte stage acts as an initiating switch-like event that sets into motion a series of changes needed for developmental progression (Fig. 8). Molecularly, how the regulated nuclear entry of VCP occurs at this developmental stage is unknown, but it appears to be controlled at some level by upstream tTAFs. Although tTAFs can bind and recruit transcriptional machinery important for the expression of spermatocyte and spermatid differentiation genes (Chen et al., 2005, 2011; Hiller et al., 2004), direct binding between tTAFs and VCP has not been documented and seems unlikely given that VCP (Fig. 2) and tTAFs (Fig. S3) (Chen et al., 2005) have distinct localization patterns. Alternatively, other indirect methods of regulation may be at play. Structural studies have indicated that phosphorylation of VCP at a C-terminal tyrosine residue supports its nuclear translocation in other systems (Madeo et al., 1998; Song et al., 2015), raising the possibility that kinase signaling, perhaps downstream of tTAFs, could also feed into this regulation. In the future, it will be important to design and characterize *VCP* mutants that cannot re-localize to the spermatocyte nucleus to probe further the molecular repercussions of this specific developmental event. Notably, impairments to VCP nucleocytoplasmic shuttling are characteristic of degenerative pathologies, including MSP-1 (Song et al., 2015), and it remains possible that similar factors may directly regulate VCP nuclear entry in somatic and germ cells. Thus, clarifying controls on VCP nuclear translocation during spermatogenesis may not only identify entry points for treating forms of male infertility, including non-obstructive azoospermia, but may also reveal novel targets for treating VCP-related degenerative diseases.

Following nuclear entry, VCP then executes molecular functions important for spermatocyte gene expression. One important function of nuclear VCP appears to be downregulation of H2Aub, an activity that supports spermatocyte differentiation and the expression of at least some tTAF-regulated genes (Fig. 8). It is important to note that although VCP is essential for male germ-cell development, it may control only one branch of signaling downstream of tTAFs; whereas blocking H2Aub in the absence of VCP permitted spermatocyte differentiation, blocking H2Aub in the absence of a tTAF, Sa, did not, consistent with previous studies (Chen et al., 2005, 2011). This suggests that tTAFs support spermatocyte differentiation through multiple signaling branches, which may be independently required. Indeed, tTAFs have been shown to also control other epigenetic modifications, including trimethylation of H3K4 (Chen et al., 2005), hinting at possible branches besides VCP-H2Aub that may act in concert.

Mechanistically, our data suggest that VCP promotes H2Aub downregulation in spermatocytes at least in part via H2A extraction and turnover. Further investigation of whether and/or how endogenous H2A turnover is developmentally coordinated among nuclei within a cyst, and if there are differences in the timing or execution of this event between cysts in the same testis, could reveal potential complexities in regulation pertinent to gene-expression control at the spermatocyte stage. Because PRC1 catalyzes H2Aub at target gene loci (Blackledge et al., 2020; de Napoles et al., 2004; Endoh et al., 2008; Wang et al., 2004), our findings also support the model that PRC1 activity is indeed inhibitory to spermatocyte gene expression and differentiation, a point of contention in the spermatogenesis field (Chen et al., 2005, 2011; El-Sharnouby et al., 2013). Interestingly, the PRC1 components Pc and Sce are still present in spermatocytes (Chen et al., 2005), where H2Aub levels are normally lowered. Thus, it is unclear how spermatocytes prevent mono-ubiquitylation of new H2A in spermatocytes. Because VCP is absent from nucleoli (Fig. 1F) and less abundant at autosomes (Fig. 1G,H), where PRC1 components localize (Chen et al., 2005), it seems unlikely that VCP would directly interact with PRC1 components outside of transient interactions, but it could potentially serve as a barrier to prevent PRC1 components from entering the nuclear interior. Alternatively, VCP may cooperate with an associated deubiquitinase, such as Asx and/or Calypso (ASXL and BAP1 in mammals, respectively) (Scheuermann et al., 2010), or extract polyubiquitylated substrates involved in making this histone modification from target gene loci, similar to how it extracts Aurora B from chromatin in *Xenopus* embryos (Ramadan et al., 2007). Consistent with the latter possibility, mutation of the major polyubiquitin gene, *Ubi-p63E*, results in a similar meiotic arrest as *VCP*-RNAi (Lu et al., 2013). Going forward, it will be important to clarify precisely how VCP downregulates H2Aub at the mitotic-meiotic transition, as this mechanism may also be pertinent to understanding the relief of PRC1 inhibition in other natural, developmental contexts.

## MATERIALS AND METHODS

### Fly husbandry and strains

Flies were maintained on standard cornmeal/agar food at 25°C, unless otherwise noted. For RNAi experiments, flies were incubated on standard cornmeal/agar food at 29°C for 5-7 days prior to dissection and imaging to boost Gal4 activity unless otherwise noted.

The following fly strains were used in this study: w^1118^, VCP-GFP (Wall et al., 2021), UAS-*VCP*-RNAi (Vienna *Drosophila* Resource Center, #24354; FBst0455418), BamGal4 (Doug Harrison, University of Kentucky, KY, USA), *sa-GFP* (Chen et al., 2005), UAS-*bam*-RNAi [Bloomington *Drosophila* Stock Center (BDSC), #33631; FBst0033631), UAS-*sa*-RNAi (BDSC, #36730; FBst0036730), UAS-*mia*-RNAi (BDSC, #57790; FBst0057790), UAS-*Sce*-RNAi (BDSC, #67924; FBst0067924), UAS-*Ufd1*-RNAi (BDSC, #41823; FBst0041823), UAS-*CG8042*-RNAi (BDSC, #41604; FBst0041604), UAS-*Faf2*-RNAi (BDSC, #43224; FBst0043224), UAS-*casp*-RNAi (BDSC, #44027; FBst0044027), UAS-*Npl4*-RNAi (BDSC, #53004; FBst0053004), UAS-*elg1*-RNAi (BDSC, #63551; FBst0063551), UAS-*Rpt2*-RNAi (BDSC, #34795; FBst0034795), *Sce^KO^* (BDSC, #80157; FBst0080157), UAS-*GFP^nls^* (lab stock), VasaGal4; hs*FLP.D5*, UAS-*GFP*/cyo; *FRT82B*, tubGal80/TM6B (Butsch et al., 2022), EyaGal4 (Leatherman and DiNardo, 2008), UASp-*FRT-H2A-eGFP*-*PolyA*-*FRT*-*H2A-mCherry* (Kahney et al., 2021), tubGal80^AID^ (McClure et al., 2022) (BDSC, #92470; FBst0092470) and UAS-*VCP^K2A^* (Chang et al., 2021).

### Fertility assay

Single male flies were placed in a vial with two or three virgin females shortly after eclosion. Males were transferred to a fresh vial with new virgin females 2-3 days later. The presence or absence of progeny was scored after each mating. Males that failed to produce progeny in both matings were scored as infertile. Males that were able to produce progeny in both matings were scored as fertile. Flies were kept at 25°C on standard cornmeal/agar food for all matings.

### Larval imaging

Third instar larvae were washed several times in PBS (137 mM NaCl, 2.7 mM KCl, 10 mM Na_2_HPO_4_, 1.8 mM KH_2_PO_4_) to remove food from larvae and prevent potential imaging artifacts. After washing, larvae were placed on a 4% agarose gel pad on a glass microscope slide and subsequently immobilized by firmly pressing a glass coverslip down on the larvae. Imaging was performed on a Leica M165 fluorescence stereomicroscope equipped with a GFP filter and 488 nm laser.

### Immunostaining, microscopy, and image processing

Testis immunohistochemistry was performed using standard procedures. Testes were dissected in PBS and then immediately fixed in 4% paraformaldehyde for 20 min. Testes were washed three times in PBT (0.1% Tween-20 in PBS), then incubated in blocking buffer (3% bovine serum albumin in PBS) for at least 1 h at room temperature. Testes were incubated with the primary antibody diluted in blocking buffer containing 2% Triton X-100 overnight at 4°C. The next day, testes were washed five times with PBT prior to applying the secondary antibody. Testes were incubated with the secondary antibody for at least 3 h at room temperature in the dark. After the secondary antibody solution was removed, testes were washed five times with PBT; 1 µM Hoechst 33342 (Invitrogen, H21492) was added to the first wash to stain DNA. Testes were mounted in Vectashield antifade mounting medium prior to imaging.

Phase-contrast imaging was performed on live testis squashes. Testes were dissected in PBS then stained with 1 µM Hoechst 33342 and 1:5000 MitoTracker Deep Red (Thermo Fisher Scientific, M22426) for 10 min in the dark at room temperature. Testes were then rinsed in PBS and then mounted in PBS.

The following antibodies were used in this study: rabbit anti-GFP (1:1000; Invitrogen, A21311), mouse anti-GFP (1:200; Invitrogen, A11120), rabbit anti-H2Aub (1:100; Cell Signaling Technology, 8240S), rabbit anti-VCP (1:100; Cell Signaling Technology, 2648), rabbit anti-mCherry (1:100; Invitrogen, PA5-34975), mouse anti-fibrillarin (a kind gift from Pat DiMario, Louisiana State University, LA, USA), rat anti-Pol II S2p (1:1000; Millipore, 04-1571, clone 3E10), mouse anti-LamC (1:100; Developmental Studies Hybridoma Bank, LC28.26), goat anti-rabbit 488 (1:500; Invitrogen, A11034), goat anti-rabbit Cy5 (1:500; Invitrogen, A10523), goat anti-mouse 488 (1:500; Invitrogen, A11001), goat anti-mouse Cy5 (1:500; Invitrogen, A10524) and goat anti-rat 568 (1:500; Invitrogen, A11077).

Images were acquired using an inverted Leica SP8 confocal microscope, equipped with a 40× oil immersion objective (NA 1.30), 40× phase contrast objective (NA 0.80) and a white-light laser. Images were processed using Leica LAS X software, and quantifications were performed using Fiji (NIH) on 8-bit images prior to adjusting brightness or contrast.

### VCP-GFP quantification

We used Fiji (NIH) to quantify VCP-GFP intensity in the nucleus and cytosol of germ cells at various stages of spermatogenesis (see below for stage identification information). Briefly, we outlined the nucleus and a small region within the cytosol of five cells per stage per testis (see figure legends for total cell and testis counts) and measured the mean fluorescence intensity. VCP-GFP was imaged using a 488 nm excitation laser and a Hybrid detector set at 500-550 nm. The excitation laser was set at 2.5% for all testes imaged with gain left at its default level, and, thus, GFP intensity can be reliably compared between genotypes. For presentation purposes, the mean intensity of the control group was set to one. We used these same methods to quantify VCP-GFP intensity in the nuclear interior and at autosomes by outlining the nuclear interior (Hoechst negative) and Hoechst-positive chromatin. In this case, the mean intensity of Hoechst-positive chromatin was set to one for presentation purposes.

### Pol II S2p quantification

We quantified Pol II S2p intensity at autosomes using Fiji by outlining autosomes and measuring the mean intensity. For presentation purposes, the mean intensity of control autosomes was set to one.

### Germ-cell stage identification

Germ-cell staging was performed primarily based on chromatin morphology. Spermatocytes were identified based on chromatin features described by Cenci et al. (1994). Spermatids were identified based on chromatin and mitochondrial features described by Fabian and Brill (2012).

### Western blotting

Total protein was extracted from 40 pairs of testes per genotype. Testes were dissected in NP-40 lysis buffer containing protease inhibitors (6 mM Na_2_HPO_4_, 4 mM NaH_2_PO_4_, 1% NP40, 150 mM NaCl, 2 mM EDTA, 50 mM NaF, 0.1 mM Na_3_VO_4_, 4 µg/ml leupeptin, one Roche cOmplete^TM^ protease inhibitor tablet per 10 ml, pH 7.4). After dissections were complete, lysis buffer was removed, and testes were further lysed in 5× SDS sample buffer (1 M Tris-HCl, pH 6.8, 1 mM DTT, 20% SDS, 60% glycerol, Bromophenol Blue) using a sterile pestle. Samples were boiled at 100°C for 10 min, followed by a brief vortex and centrifugation at 20,000 ***g*** for 1 min. Proteins were resolved on a 4-12% Bis-Tris gel and transferred to a nitrocellulose membrane. Membranes were blocked in 4% milk for at least 1 h. After blocking, membranes were incubated overnight with primary antibody at 4°C. The next day, membranes were washed three times for at least 10 min in PBT. Membranes were then incubated with secondary antibody for 1 h at room temperature, followed by washing. Membranes were treated with an ECL chemiluminescent reagent (Bio-Rad) and proteins were visualized on a Bio-Rad ChemiDoc imaging system.

The following antibodies were used in this study: rabbit anti-H2Aub (1:2000; Cell Signaling Technology, 8240S), mouse anti-Actin (1:1000; Invitrogen, MA5-11869), goat anti-rabbit IgG HRP (Invitrogen, 31460) and goat anti-mouse IgG HRP (Invitrogen, 31430).

### RT-qPCR

Total RNA was extracted from 50 pairs of testes per genotype by Trizol extraction. Total RNA was treated with DNase (Promega, M610A) to remove genomic DNA, followed by RNA precipitation with isopropanol and washes with 70% ethanol. Five-hundred nanograms of RNA was used for cDNA synthesis using the iScript cDNA synthesis kit (Bio-Rad, 1708891). qPCR was conducted using PowerUp^TM^ SYBR^TM^ Green Master Mix (Thermo Fisher Scientific). Data were analyzed using Design and Analysis software (v2.6; Thermo Fisher Scientific). The 2^−ΔΔCt^ method was used to estimate the relative changes in gene expression. Primers used for each gene were: *Actin 5C* forward TTGTCTGGGCAAGAGGATCAG, reverse ACCACTCGCACTTGCACTTTC; *Cyclin A* forward GAAAGTTCAGGTCCTTCCGTGAC, reverse GGGCACCACATTCGATTTCT; *janB* forward CTCCGTTTCAAAAATGTTACTCAACCG, reverse CAACATCGGCGCCACG; *gdl* forward GAACTTCCCGAAAACTTGCAGACAC, reverse CGTTCTCCGCCTGCATCC; *Mst35Bb* forward GTGGAATGGCATAATTTCCATTTCTGC, reverse GTTCACTGGTGGTGACCTTGC; *Mst98C* forward GCGGTCCTTGTAGTCCATGC, reverse CTCCGGCACAATCTTCTCCG; *Mst87F* forward TCCGACTTGTCAAACCGATA, reverse GCACGAAGGGTATCCACAAT; *Ubi-p63E* forward GGCTAAGATCCAAGACAAGGAA, reverse GAGACGAAGGACCAAGTGAAG; *Hsc70-2* forward GAACCAGGTGGCCATGAAT, reverse TCAGGTCCTCCTGTATCTTCTT; *Cyt-c-d* forward CCTCAAGGACCCGAAGAAATAC, reverse TGACTTGAGGAAGGCAATCAA; *HtrA2* forward CTATCCGATGGCAGGACTTT, reverse CCGAAAGATTGTTCACCTGTATG; *ACXC* forward GGAACACAGTTACTTGAGGGAAA, reverse CATCAGACGGACCTGGTAGTA; *kl-3* exon 2 forward CCCGAGCATTTAATAACCACAAG, reverse AACGGACATTATCCTTAGCTTCA; *kl-3* exon 6 forward GCTGGATCTAAGAGGTCATTGG, reverse GGCTGAATGTAACACCCGTTAT; *kl-3* exon 14 forward TATGTCCATTCAACCTAAAGAATCGTC, reverse CCCATTGCAATTAGATGCTGTT.

### MARCM

VasaGal4; hs*FLP.D5*, UAS-*GFP*/UAS-*VCP*-RNAi; *FRT82B*, *Sce^KO^*/*FRT82B*, tubGal80 and VasaGal4; hs*FLP.D5*, UAS-*GFP*/UAS-*VCP*-RNAi; *FRT82B*/*FRT82B*, tubGal80 males were generated by standard crossing procedures (Butsch et al., 2022). Males were heat shocked at 37°C the same day they eclosed for 1 h to activate FLP expression and drive recombination. Following heat shock, flies were housed at 25°C on standard agar/cornmeal food until dissection. Testes were dissected and imaged at the indicated time points following heat shock (‘clone induction’).

### H2A turnover assay and PCR verification

hs*FLP.D5*/+; BamGal4/UASp-*FRT-H2A-eGFP*-*PolyA*-*FRT-H2A-mCherry* and hs*FLP.D5*/UAS-*VCP*-RNAi; BamGal4/UASp-*FRT-H2A-eGFP*-*PolyA*-*FRT-H2A-mCherry* males were generated by standard crossing procedures. Males were heat shocked at 37°C the same day they eclosed for 1 h to activate FLP expression and drive removal of the *H2A-eGFP* cassette. Following heat shock, flies were housed at 25°C on standard agar/cornmeal food for 5 days until dissection, antibody labeling, and imaging. We quantified H2A-GFP and H2A-mCherry intensity by outlining testes and measuring the mean GFP and mCherry intensities.

We verified the recombination efficiency by performing PCR using a forward primer against UASp (GCTTGTTGAGAGGAAAGGTTGTGTGCG) and a reverse primer against mCherry (GGTGGTCTTGACCTCAGCGTCG). Primers were annealed at 70°C with a 3.5 min extension time for 30 cycles.

### Auxin-induced temporal expression of *VCP^K2A^*

VasaGal4; tubGal80^AID^ flies were generated by standard crossing procedures and subsequently crossed to UAS-*VCP^K2A^* flies to generate VasaGal4; UAS-*VCP^K2A^*/+; tubGal80^AID^/+ males. Males were fed 5 mM auxin or standard food (see above) at 29°C for 10 days prior to dissection and imaging. Flies were transferred to fresh food every 2-3 days.

### Statistical analyses

Information on sample size and statistics is provided in figure legends where applicable. Data normality was tested via the D'Agostino-Pearson test in combination with Q-Q plots prior to performing follow-up statistical analyses using GraphPad Prism software. Statistical tests used to determine significance are indicated in figure legends. The Student's unpaired *t*-test was used when unpaired data for two groups were normally distributed and standard deviation was equal between both groups. Welch's unpaired *t*-test was used when unpaired data for two groups were normally distributed, but standard deviation was not equal. The Mann–Whitney U-test was used when unpaired data for two groups were not normally distributed. The paired *t*-test was used when paired data for two groups were normally distributed and standard deviation was equal. The Wilcoxon matched pairs signed rank test was used when paired data for two groups were not normally distributed. The Brown–Forsythe ANOVA with Dunnett's multiple comparisons test was used when there were more than two groups with normally distributed data, but standard deviation was not equal between the groups.

## Supplementary Material

10.1242/develop.201557_sup1 Supplementary information
