# Supplementary material for: VCP promotes tTAF-target gene expression and spermatocyte differentiation by downregulating mono-ubiquitylated H2A
Source: Development. 2023 Jul 19;150(14):dev201557. doi: 10.1242/dev.201557 (PMC10399981; doi:10.1242/dev.201557)
Supplement: Supplementary information [file develop-150-201557-s1.pdf]

Figure S1

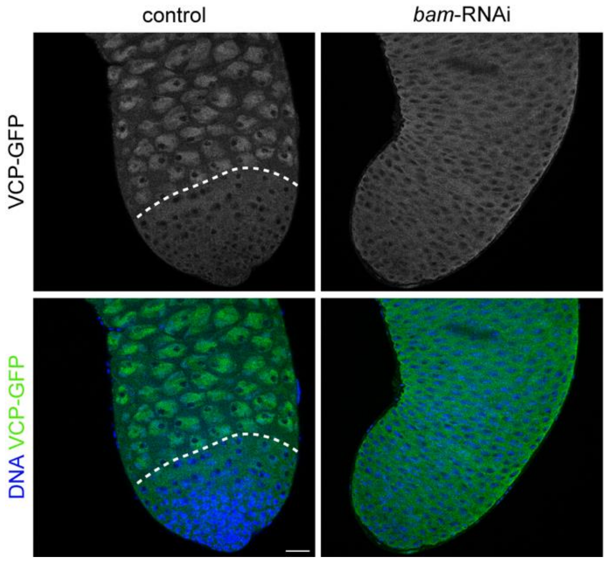

Fig. S1. VCP is cytosolic in spermatogonia, but nuclear in spermatocytes. (**A**) Images of Hoechst (DNA) and VCP-GFP in control (BamGal/+) and *bam*-RNAi (BamGal>*bam*-RNAi) testes. The dashed line indicates the mitotic-meiotic transition. Bar, 20 µm.

Development • Supplementary information

Figure S2

A

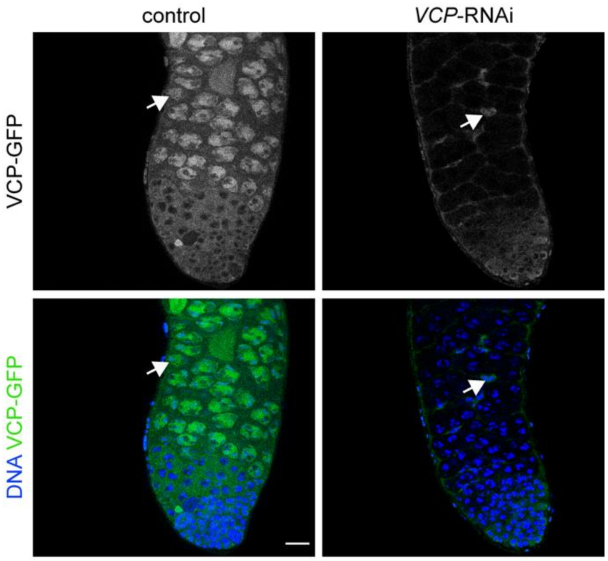

B

male fertility

control	*VCP*-RNAi
22/23	0/17

Fig. S2. Germline-specific knockdown of VCP causes male infertility. (**A**) Images of Hoechst (DNA) and VCP-GFP in control (BamGal/+) and *VCP*-RNAi (BamGal>*VCP*-RNAi) testes. The arrows indicate cyst cells. Bar, 20 µm. (**B**) Table of the proportion of males that are fertile.

Development • Supplementary information

Figure S3

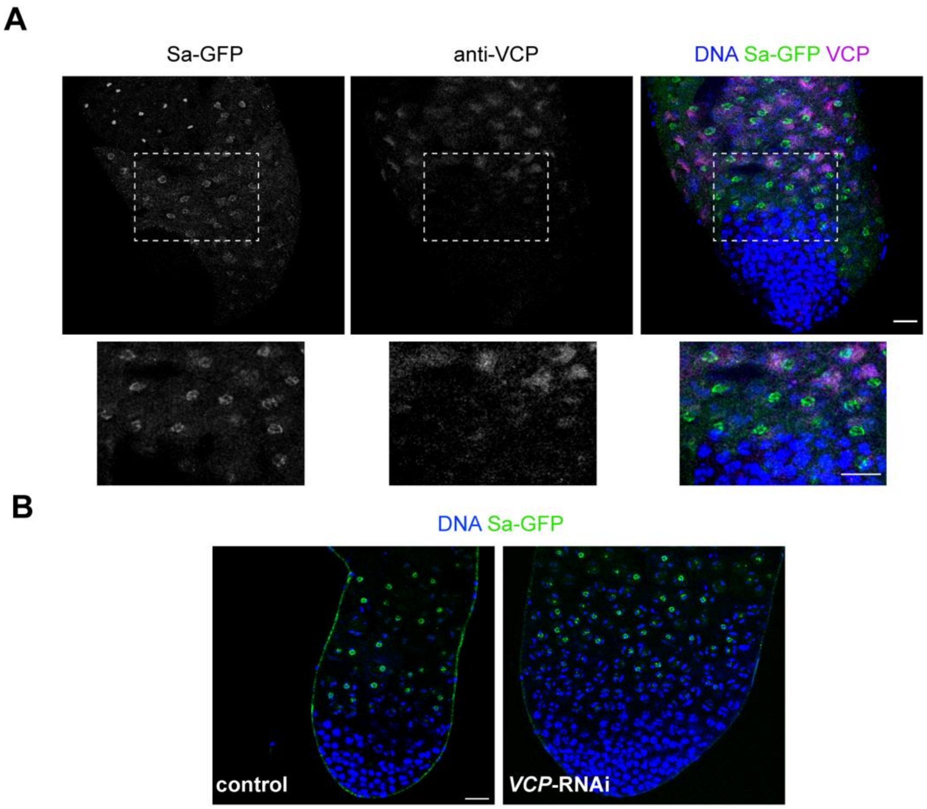

Fig. S3. VCP acts downstream of tTAFs. (**A**) Images of Sa-GFP, VCP (anti-VCP), and Hoechst (DNA) in adult testes. Outlines indicate the region shown in the insets below. (**B**) Images of Sa-GFP and Hoechst (DNA) in control (BamGal/+) and *VCP*-RNAi (BamGal>*VCP*-RNAi) testes. Bars, 20 µm.

Development • Supplementary information

Figure S4

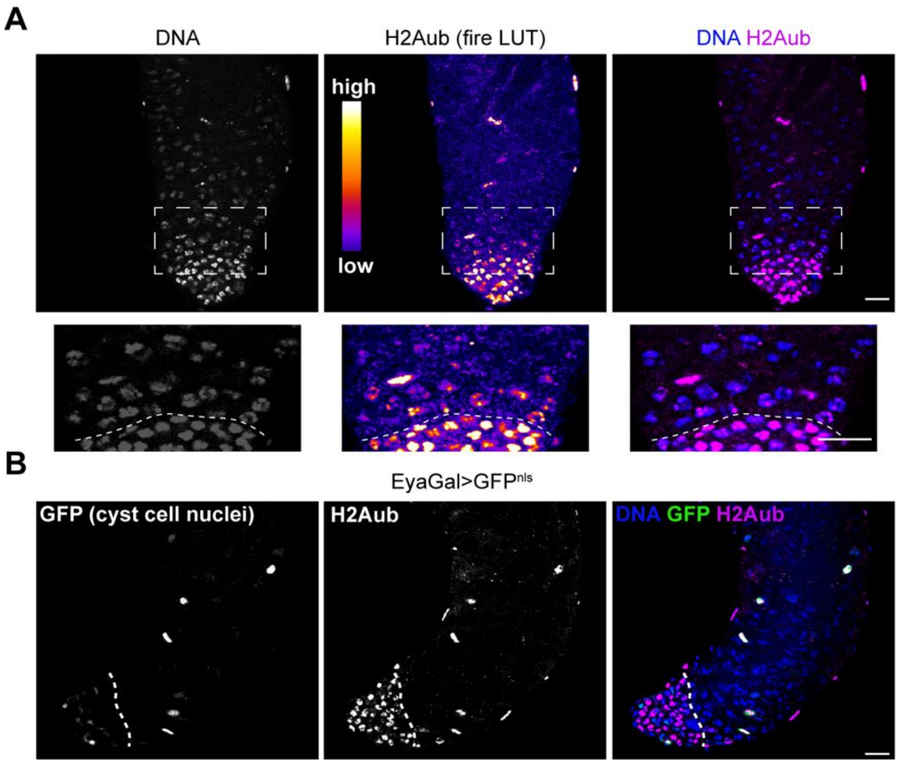

Fig. S4. H2Aub is downregulated in germ cells at the mitotic-meiotic transition but not in somatic cyst cells. (**A**) Images of H2Aub and Hoechst (DNA) in wild type (w^{1118}) testes. A heatmap image of H2Aub is presented in the middle panel. Outlines indicate the region shown in the insets below. The dashed line indicates the mitotic-meiotic transition. (**B**) Images of GFP (cyst cell nuclei), H2Aub, and Hoechst (DNA) in a EyaGal>GFP^{nls} testis. The dashed line indicates the mitotic-meiotic transition. Bars, 20 µm.

Development • Supplementary information

Figure S5

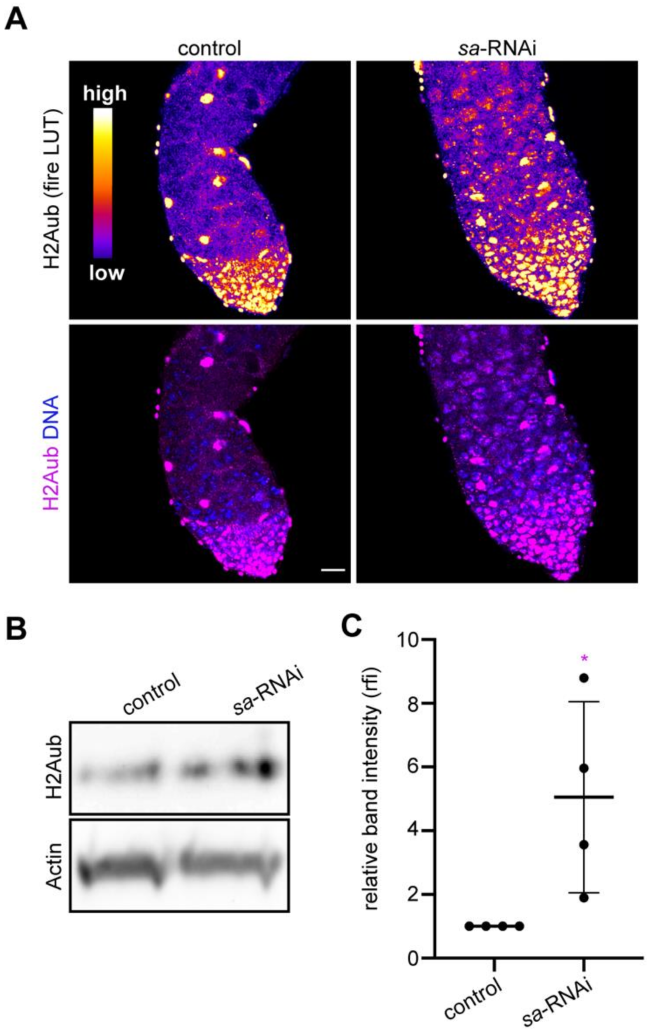

Fig. S5. The tTAF, Sa, promotes H2Aub downregulation in spermatocytes. (**A**) Top panel: Heatmap images of H2Aub in control (BamGal4/+) and *sa*-RNAi (BamGal4>*sa*-RNAi) testes. Bottom panel: Fluorescence images of H2Aub and Hoechst (DNA) in control (BamGal4/+) and *sa*-RNAi (BamGal4>*sa*-RNAi) testes. Bar, 20 µm. (**B**) Western blotting for H2Aub (top) and Actin (bottom, loading control) in control and *sa*-RNAi testes. (**C**) Quantification of H2Aub band intensity normalized to Actin band intensity (*n*= 4 replicates). Mean ± s.d. *, $p<0.05$. Unpaired t-test.

Figure S6

A

gene	↑H2Aub when KD?
Ufd1	yes
elg1	no
casp	no
Npl4	no
Faf2	no
CG8042	no

B

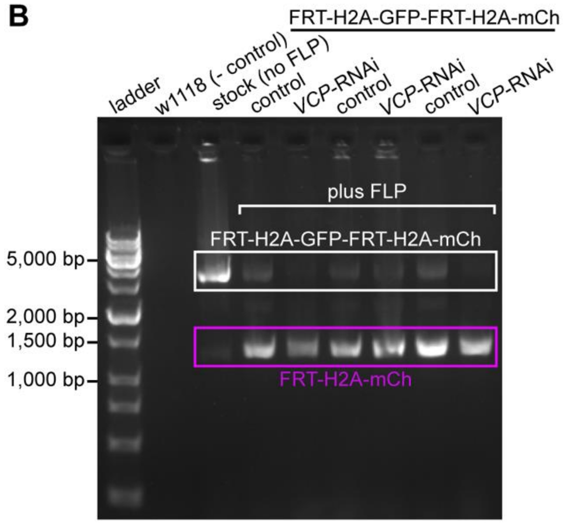

C

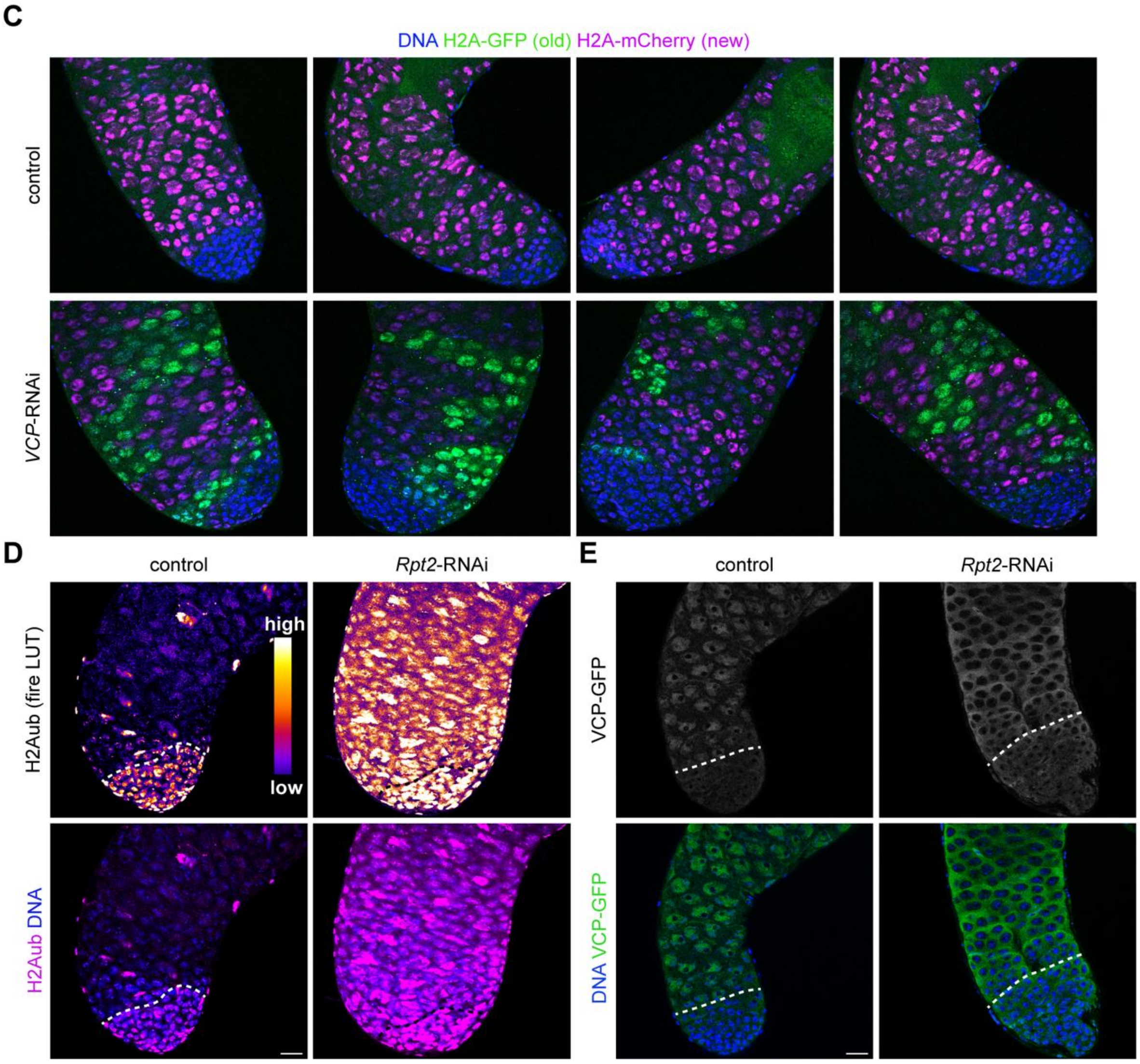

Development • Supplementary information

Fig. S6. Tested VCP cofactors, verification of FLPase-mediated recombination, and the effect of proteasome inhibition on H2Aub downregulation and VCP nuclear entry. (**A**) Table of known VCP interactors that were tested as part of the small-scale RNAi screen for H2Aub regulators. Whether H2Aub was increased after gene knockdown (KD) is indicated. (**B**) Gel image showing bands of the entire H2A turnover transgene (FRT-H2A-GFP-FRT-H2A-mCh; top band) and the partial H2A-mCherry cassette formed after FLPase recombination (FRT-H2A-mCh; bottom band). Samples from testes with the FLP transgene are indicates. PCR was performed on DNA extracted from the indicated genotypes. (**C**) Images of Hoechst (DNA), H2A-GFP (old H2A), and H2A-mCherry (new H2A) in control (BamGal/+) and *VCP*-RNAi (BamGal>*VCP*-RNAi) testes. Testes were dissected and imaged five days after FLPase induction (H2A-GFP cassette removal). (**D**) Top panel: Heatmap images of H2Aub in control (BamGal/+) and *Rpt2*-RNAi (BamGal>*Rpt2*-RNAi) testes. Bottom panel: Fluorescence images of H2Aub and Hoechst (DNA) in control (BamGal/+) and *Rpt2*-RNAi (BamGal>*Rpt2*-RNAi) testes. The dashed lines indicate the mitotic-meiotic transition. Note that the dashed lines are different colors (white in control, black in *Rpt2*-RNAi) strictly to make the line more visible in both sets of images. (**E**) Images of VCP-GFP and Hoechst (DNA) in control and *Rpt2*-RNAi (BamGal>*Rpt2*-RNAi) testes. The dashed lines indicate the mitotic-meiotic transition. Bars, 20 µm.

Development • Supplementary information

Figure S7

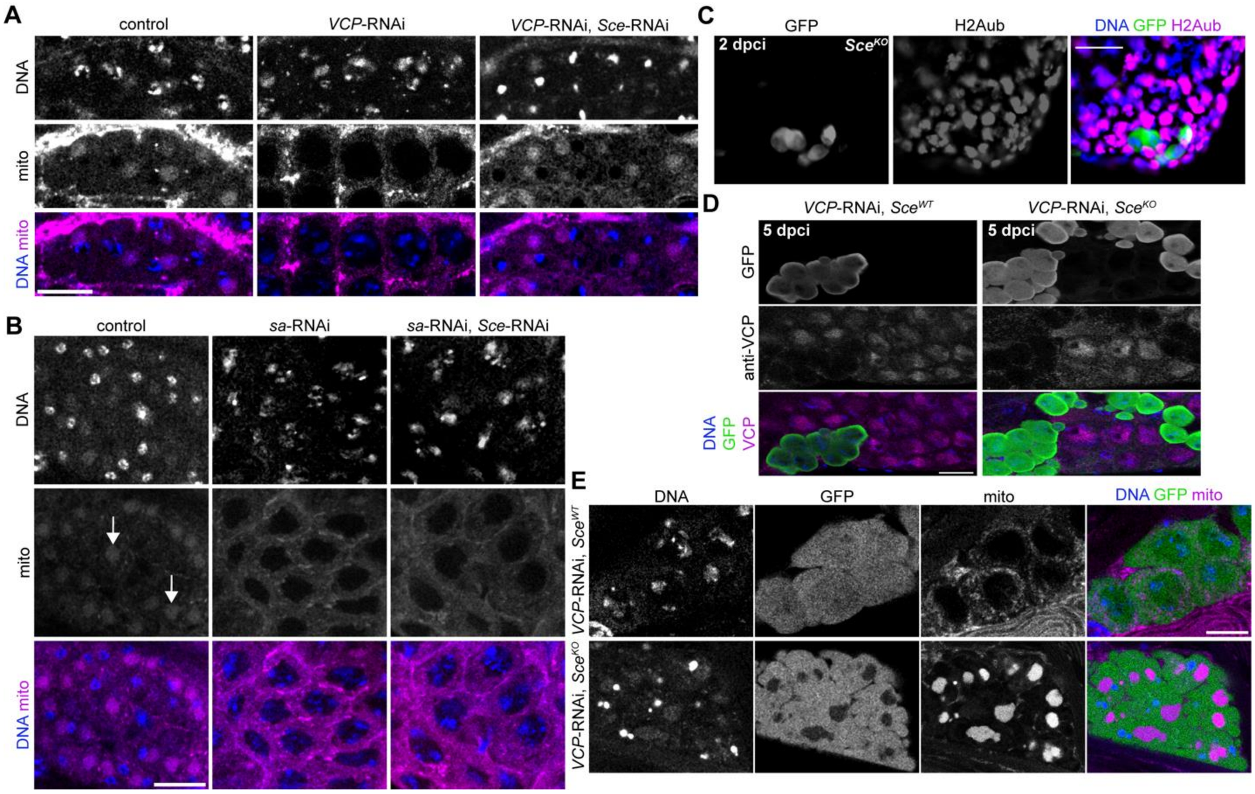

Fig. S7. Supplementary analyses of germ-cell biology after inhibition of *Sce* function by RNAi or MARCM. (**A**) Images of MitoTracker and Hoechst (DNA) in control (BamGal/+), *VCP*-RNAi (BamGal>*VCP*-RNAi), and DKD (BamGal>*VCP*-RNAi, *Sce*-RNAi) testes. Arrows indicate example nebenkerns, markers of the round spermatid stage only present in post-meiotic germ cells. (**B**) Images of MitoTracker and Hoechst (DNA) in control (BamGal/+), *sa*-RNAi (BamGal>*sa*-RNAi), and *sa*-RNAi, *Sce*-RNAi (BamGal>*sa*-RNAi, *Sce*-RNAi) testes. The most developed germ cells in *sa*-RNAi and *sa*-RNAi, *Sce*-RNAi testes were spermatocytes (note the presence of bivalents and the absence of a nebenkern). Round spermatids are shown in control testes for comparison (note the compact chromatin morphology and the nebenkern [arrows]). (**C**) Images of GFP (*Sce*^{KO} MARCM clones), H2Aub, and Hoechst (DNA) in spermatogonia at 2 dpci. (**D**) Images of GFP (MARCM clones), VCP (anti-VCP), and Hoechst (DNA) in spermatocytes at 5 dpci. In the *VCP*-RNAi, *Sce*^{WT} panel, GFP-positive MARCM clones express *VCP*-RNAi and are *Sce*^{WT} homozygotes. In the *VCP*-RNAi, *Sce*^{KO} panel, GFP-

Development • Supplementary information

positive MARCM clones express *VCP*-RNAi and are *Sce*^{KO} homozygotes. (**E**) Images of GFP (MARCM clones), Hoechst (DNA), and MitoTracker in testes at 8 dpci. Spermatocytes are shown in the *VCP*-RNAi, as indicated by the large cell size, dim Hoechst labeling, separation of bivalents, and punctate mitochondria. Spermatids are shown in the *VCP*-RNAi, *Sce*^{KO} panel, indicated by the small cell size, bright Hoechst staining, compact chromatin, and large, clustered mitochondria adjacent to nuclei. Bars, 20 µm.
