# Supplementary material for: VCP promotes tTAF-target gene expression and spermatocyte differentiation by downregulating mono-ubiquitylated H2A
Source: Development. 2023 Jul 19;150(14):dev201557. doi: 10.1242/dev.201557 (PMC10399981; doi:10.1242/dev.201557)
Supplement: Supplementary file 1 [file develop-150-201557_review_history.pdf]

VCP promotes tTAF-target gene expression and spermatocyte differentiation by downregulating mono-ubiquitylated H2A

Tyler J. Butsch, Olga Dubuisson, Alyssa E. Johnson and K. Adam Bohnert
DOI: 10.1242/dev.201557

Editor: Cassandra Extavour

Review timeline
Original submission: 21 December 2022
Editorial decision: 30 January 2023
First revision received: 26 April 2023
Accepted: 26 June 2023

Original submission

First decision letter

MS ID#: DEVELOP/2022/201557

MS TITLE: VCP acts downstream of tTAFs to downregulate mono-ubiquitinated H2A and promote spermatocyte differentiation in Drosophila

AUTHORS: Tyler Butsch, Olga Dubuisson, Alyssa E. Johnson, and K. Adam Bohnert

I have now received all the referees' reports on the above manuscript, and have reached a decision. The referees' comments are appended below, or you can access them online: please go to BenchPressand click on the 'Manuscripts with Decisions' queue in the Author Area.

As you will see, the referees express considerable interest in your work, but have some significant criticisms and recommend a substantial revision of your manuscript before we can consider publication. If you are able to revise the manuscript along the lines suggested, which may involve further experiments, I will be happy receive a revised version of the manuscript. Your revised paper will be re-reviewed by one or more of the original referees, and acceptance of your manuscript will depend on your addressing satisfactorily the reviewers' major concerns. Please also note that Development will normally permit only one round of major revision. If it would be helpful, you are welcome to contact us to discuss your revision in greater detail. Please send us a point-by-point response indicating your plans for addressing the referee's comments, and we will look over this and provide further guidance.

Please attend to all of the reviewers' comments and ensure that you clearly highlight all changes made in the revised manuscript. Please avoid using 'Tracked changes' in Word files as these are lost in PDF conversion. I should be grateful if you would also provide a point-by-point response detailing how you have dealt with the points raised by the reviewers in the 'Response to Reviewers' box. If you do not agree with any of their criticisms or suggestions please explain clearly why this is so.

© 2023. Published by The Company of Biologists under the terms of the Creative Commons Attribution License (https://creativecommons.org/licenses/by/4.0/).

Reviewer 1

Advance summary and potential significance to field

This is a very nice study of the interaction between specific transcriptional machinery elements (tTAFs) and chromatin (H2Aub), indicating that VCP mediated replacement of H2Aub is required for the tTAFs to function properly. The authors clearly show that VCP-dependent H2Aub modulation is essential for the male germline transition into meiosis. The transition from spermatagonia to spermatocytes is characterized by a truly incredible transcriptional increase enlargement of the cells, activation of the Y-chromosome, and inactivation of the X.

The authors constructed a well-written story that ramps up to a crescendo in figure 5 and 6. The images are beautiful, results are quantified, and methods are clear. Those of us in the spermatogenesis field will certainly want to read and study this manuscript. There are also implications for anyone interested in transcription and chromatin.

Comments for the author

Major
1) My only significant scientific criticism is related to subnuclear structure and the relationship with gene expression. Basically, I became interested enough to want more. Many images are too small to get the details of the critical spermagonia-spermatocyte transition (Figures 3 and 5 especially Methods on staging). This need not require new experiments, although that might be warranted based on the findings:

a. It looks like the nuclei enlarge in VCP mutants. How much? And how does this compare to wt? The question here is whether nuclear volume increase requires the transcriptional upregulation of the spermatocyte gene battery.
b. The images are too small, but it looks like the nuclei have well developed nuclear territories. Do they? What do they look like? When I zoomed in, I thought the VCP nuclei looked odd. Is the X condensed and inactivated? Are autosomal territories condensed or diffuse? Is the Y active? Is the nucleolus well formed?
c. The H2Aub staining does not appear to fully overlap DAPI in the GCs but it does appear to fully overlap in somatic cyst cells, but it's hard to tell at the current resolution. A zoomed in view of single nuclei would be nice. DAPI dark versus DAPI light? Territories?

2) The authors should cite and discuss some of the relevant single cell data as it pertains to the sharp expression transition e.g. PMID: 33563972 PMID: 34403408 PMID: 35239393

3) "Available on request" does not hack it anymore. Please have the authors deposit all relevant data in a repository. These exist for virtually all data types. See Figshare, Open Science, Zenodo, etc. Development may have preferences.

4) Thank the authors for using stock center ids. Using shorthand for alleles and lines creates confusion in the literature and overtaxes already overextended FlyBase staff. Please take one more step and use FlyBase identifiers and preferably use the FlyBase Alleles and Reagent Table (ART). https://wiki.flybase.org/wiki/FlyBase:Author_Reagent_Table_(ART).

Minor
5) It seems to me that VCP and tTAFs are required for the transcriptional program. I'd play with the title a bit, along the lines of Figure 7.

6) 110: "stem cells, spermatogonia, and spermatocytes" or the like, would be better than "mostly spermatocytes".

7) The Authors could be a bit bolder is some areas, for example, state explicitly what rescue of VCP by H2Aub KD means. Readers are busy, you have to hit them over the head when you have a critical result to explain.

© 2023. Published by The Company of Biologists under the terms of the Creative Commons Attribution License (https://creativecommons.org/licenses/by/4.0/).

8) I enjoyed most of the supplemental figures and think that the authors might be able to incorporate some of their favorites into the smallish main figures. If they want to make room, the later stages of spermatogenesis might not be needed in the main figures, given the spermatocyte block.

9) The particular drivers and antibodies have been well described, but it is always nice to state that the patterns match expectations. It never hurts to do at least a few quick experiments with additional drivers.

Reviewer 2

Advance summary and potential significance to field

In their manuscript, Butsch and coauthors investigate the role of Valosin-containing protein (VCP) in the Drosophila male germline. They demonstrate that VCP is cytoplasmic in mitotic spermatogonia and translocates to the nucleus in meiotic spermatocytes. In turn nuclear translocation of VCP in primary spermatocytes is dependent on testis-specific TBP-associated factors (tTAF) and is required to drive spermatocyte differentiation. Indeed VCP-RNAi testes do not contain germ cells that develop beyond the spermatocyte stage. They further show that nuclear VCP is required for downregulating a repressive histone modification mono-ubiquitinated H2A (H2Aub), leading to spermatocyte differentiation.

Remarkably, inhibiting PRC1 ubiquitin ligase activity in the absence of VCP, is sufficient to overcome the meiotic-arrest phenotype and to promote development through meiosis, indicating that H2Aub downregulation is an important function of VCP in spermatocyte differentiation. This manuscript provides a significant contribution to our understanding of the mechanisms underlying Drosophila spermatogenesis. Thus, this paper is suitable for publication in Development.

Comments for the author

I suggest minor issues that the authors need to address before the manuscript can be accepted.
1) The sentence in the abstract (lines 30-32): “Like tTAF mutants spermatocyte gene expression fails to properly activate in VCP-RNAi testes, and germ cells arrest in early meiosis” is not clear and should be rephrased.
2) There are too many supplemental figures. On the other hand, some figures contain few panels. I think that the authors can reorganize the figures to show essential information in the main figures.
3) The panels Figure 4 and Figure S5 show western blots of poor quality.
Overloading of loading control (actin) makes it difficult to quantify the difference between the mutant and the control.
4) In Figure 4D it is difficult to appreciate the difference in H2Aub intensity.
5) The authors say: “Combined knockdown of VCP and sce allowed germ cells to develop beyond the spermatocyte stage to the round spermatid stage, indicated by compact chromatin morphology and accumulation of mitochondria in a nebenkern structure (Fabian and Brill, 2012) adjacent to germ-cell nuclei (Fig. 6D).” It is weird to show the nebenkern by using mitotracker staining of mitochondria. Could it be possible to show phase contrast images of spermatids in the three genotypes?

Reviewer 3

Advance summary and potential significance to field

Overall the paper provides and interesting advance for the field. The finding that VCP translocation to the nucleus is important for down regulation of H2Aub, for ensuring normal spermatocyte development, and for ensuring normal spermatocyte transcription are all well supported and point

© 2023. Published by The Company of Biologists under the terms of the Creative Commons Attribution License (https://creativecommons.org/licenses/by/4.0/).

to a previously unknown aspect of regulation in these cells. There are some issues with data presentation, and some areas where the logic of the experimental design was not clear to me.

Comments for the author

Introduction: As a spermatogenesis expert rather than a VCP expert, I would like just a little more information in the introduction about what this protein does molecularly, as written it is clear it is in lots of cellular processes, but not how it works. What happens to ubiquitinated cargo after VCP has bound it.

Results:
Figure 1, localisation change to nucleus in primary spermatocytes is very clear. It is also clear from the pictures that it is excluded from the nucleolus (and maybe enriched on Y-loops?). The exclusion from nucleolus is relevant to the context of PRC1 and tTAFS, since both of them are enriched in the nucleolus at this stage. This should be mentioned and commented on. For figure 1 (and later figures) it's really hard to see the DNA when shown in blue. And the single channel images should be presented in grayscale throughout. (https://www.youtube.com/watch?v=JT9mUkEG-C0) explains why.

RNAi experiments. It's worth reminding the reader that null mutants are lethal, and that the gene is essential for cell viability. Fig S2, would be nice to see the GFP only channel separated out and in grayscale. The protein remaining in spermatogonia and in cyst cells would be more obvious.

Figure 2. The RNAi testes are clearly much smaller than WT. It would be better to use phase contrast microscopy rather than DIC for this imaging. It is very hard to see the morphology of the spermatocytes in the RNAi testes. It is not clear if they are earlier or later spermatocyte stages as shown. The Hoechst labelling sort of helps, but again is very hard to interpret. The statement that the arrest is at the point that VCP enters the nucleus needs more data to support it. VCP seems to enter the nucleus in very early spermatocytes, but the precise arrest point seems to be later. The down regulation of tTAF target genes is clear, but a causal link is harder to make. The selected genes increase in expression as spermatocytes mature. If the RNAi causes an arrest early in spermatocytes these genes would be reduced in expression because the cells remain early, not because of a direct transcriptional effect. It would be good to test a gene expressed in primary spermatocytes that does not depend on tTAFs. An in situ hybridisation could also help confirm the reduction in the RNAi spermatocytes.

The increase in H2Aub in RNAi spermatocytes is very clear.
I am confused by the overexpression experiments with the auxin system. If the constructs are only being driven in the germline (vasaGal4), why do you need the Gal80-AID and auxin at all? Why keep the flies on auxin for 10 days? Why not use bamGal4 for this experiment? What effect does the knock out of the ATPase activity have on the molecular function of VCP, ie why is this dominant (this links back to what exactly does VCP do)?

Screen of co-factors. "we screened several… and found one…". It would be very nice to know which ones did not give you a phenotype too. The old vs new H2A experiment is intriguing, but I wonder about technical issues. The main text should state that the H2A construct is under UAS control, thus you are only assaying protein that has been made in the spermatocytes, and that is in addition to endogenous protein. The hs-flp excision is clearly very efficient in the WT testes, as all cells are red, not green. Are you sure that flp was so efficient in the mutant? In the RNAi testes there is variability between cysts, but not within cysts, how is the H2A turnover coordinated between cells within a cyst? How does the proposed inter-cyst variability in H2A turnover rate correlate with the very uniform (between cysts) up regulation of H2Aub seen in the other stainings?

Fig S8A, the labels above the middle and right hand panels are switched around. The data shown in figure S8C seems quite important to the message of the paper – why is this a supplement? I agree that the VCP-RNAi, sceKO clones do make cells that look like post-meiotic stages, but these are extremely abnormal (phase contrast would be nice here too).

I gave this manuscript to an early career researcher to comment on. Her feedback is below. (some of this overlaps with comments I have already made above)

© 2023. Published by The Company of Biologists under the terms of the Creative Commons Attribution License (https://creativecommons.org/licenses/by/4.0/).

Line 41. “ubiquitin-selective protein segregase” - I'm not sure I know what this is without more explanation.

Line 51. Stage specific transcriptomic data… Fly Cell Atlas shows it is highest in spermatocytes, also spermatogonia in and cyst cells. This correlates with your protein data.

Line 127-128 (bam-RNAi). Images look convincing. However, why do it this way? What if nuclear translocation is bam dependent? Is this really necessary if you can just count the number of spermatogonia/spermatocytes in a cyst? Surely this will tell you when translocation occurs? I would like to see some pictures of individual cysts.

Line 142-144. I know the whole testis images are meant to be at the same magnification, but a larger magnification image of the VCP-RNAi testes would be really nice. It would make the meiotic arrest phenotype easier to see.

Line 146-148. I don't find Fig 2C particularly clear. Perhaps more labels would help? Also images of cysts would make stage clearer as well.

Page 153-155. Could test this by making a mutant for which the protein doesn't localise in the nucleus, but probably not for this paper.

Page 158-160. Why test tTAFs and not also tMAC?

Line 242-244. I got a bit lost here Line 245-256. Having now read this section, I feel like this should be 'partially suppresses' or something like that. I guess the DKD aren't technically meiotic arrest phenotype anymore... but they haven't been fully rescued.

Line 303-304. I could do with a diagram.

Line 313-314 So what stage do the DKD testes get to? They mention onion stage, but any further? Any elongation at all? (Histone ubiquitination in canoe stage nuclei, do VCP-RNAi get to this stage?)

Line 423-425. Maybe I've missed it but I can't find what tool they used to measure relative fluorescence intensities for all of those comparisons between cytoplasmic and nuclear VCP-GFP in their various RNAi lines.

Line 761-763 I'm unclear whether the test was between normal and each RNAi line, or whether the two RNAi lines were also compared.

Line 786. partially rescues?

Figure 3C. Is there a sig. dif. between sa-RNAi and VCP-RNAi? If so is this because of differences in RNAi efficiency or because other stuff is involved in expression of these genes downstream of the tTAFs?

First revision

Author response to reviewers' comments

We would like to thank the reviewers for their time and helpful comments on our manuscript. We are pleased that our manuscript was positively received by all three reviewers. Reviewer 1 stated that our manuscript is a “well-written story” and that researchers in the spermatogenesis field will “certainly want to read and study this manuscript.” Reviewer 2 stated that our manuscript “provides a significant contribution to our understanding of the mechanisms underlying Drosophila spermatogenesis”. Similar to the first two reviewers, Reviewer 3 stated that our paper “provides an

© 2023. Published by The Company of Biologists under the terms of the Creative Commons Attribution License (https://creativecommons.org/licenses/by/4.0/).

interesting advance for the field.” However, each reviewer requested revisions and in some cases more data to shore up some weaker parts of the paper. Reviewer 1 requested further description of the subnuclear localization of VCP and data to show how VCP regulates nuclear morphology. Reviewer 2 requested minor text and image adjustments, along with phase contrast images to better show cell morphology. Reviewer 3 also requested further information regarding the subnuclear localization of VCP and the use of phase contrast microscopy. We have now completed revising our manuscript based on the comments from each reviewer. We believe that the added data and changes to the text and figures that were requested by reviewers have significantly strengthened our manuscript without altering any of the main conclusions. Below we provide a detailed description of how we addressed each point.

Reviewer 1 Advance Summary and Potential Significance to Field:
This is a very nice study of the interaction between specific transcriptional machinery elements (tTAFs) and chromatin (H2Aub), indicating that VCP mediated replacement of H2Aub is required for the tTAFs to function properly. The authors clearly show that VCP-dependent H2Aub modulation is essential for the male germline transition into meiosis. The transition from spermatagonia to spermatocytes is characterized by a truly incredible transcriptional increase, enlargement of the cells, activation of the Y-chromosome, and inactivation of the X.

The authors constructed a well-written story that ramps up to a crescendo in figure 5 and 6. The images are beautiful, results are quantified, and methods are clear. Those of us in the spermatogenesis field will certainly want to read and study this manuscript. There are also implications for anyone interested in transcription and chromatin.

Reviewer 1 Comments for the Author: Major
1)My only significant scientific criticism is related to subnuclear structure and the relationship with gene expression. Basically, I became interested enough to want more. Many images are too small to get the details of the critical spermagonia-spermatocyte transition (Figures 3 and 5 especially, Methods on staging). This need not require new experiments, although that might be warranted based on the findings:

a.It looks like the nuclei enlarge in VCP mutants. How much? And how does this compare to wt? The question here is whether nuclear volume increase requires the transcriptional upregulation of the spermatocyte gene battery.

Thank you for the observation; yes, this is correct. To quantify whether nuclei are enlarged, we used an antibody against Lamin C to label the nuclear envelope in control and *VCP*-RNAi testes and measured the nuclear area of stage-matched control and *VCP*-RNAi spermatocytes. Interestingly, nuclear area was significantly larger in *VCP*- RNAi spermatocytes compared to controls (Fig. 2F, G; lines 172-177). However, the nuclear swelling phenotype may not be directly linked to the transcriptional defects observed in *VCP*-RNAi testes, as nuclear area was still enlarged when we blocked H2Aub in the absence of VCP (Fig 6E, F; lines 360-363). Given that VCP functions to drive the extraction and degradation of other proteins more generally, this phenotype may be due to a general accumulation of several different proteins (i.e., not just histones) in the nucleus. There is in fact evidence that failure in VCP-dependent extraction of other proteins causes nuclear swelling in *Drosophila* photoreceptors (Chang et al., 2021. *Nat Comm*). Apparently, though, extraction of H2Aub is the critical event for tTAF-target gene expression in the testis. In sum, while VCP-mediated downregulation of H2Aub in spermatocytes drives transcriptional upregulation of tTAF-target genes (Fig. 6G), it does not lead to downsizing of the nucleus back to the normal size. Thus, in this system, VCP-dependent control of nuclear size and transcription of tTAF-target genes may be uncoupled.

b.The images are too small, but it looks like the nuclei have well developed nuclear territories. Do they? What do they look like? When I zoomed in, I thought the VCP nuclei looked odd. Is the X condensed and inactivated? Are autosomal territories condensed or diffuse? Is the Y active? Is the nucleolus well formed?

Thank you for the suggestion. We have tried to include appropriately sized images throughout the revised manuscript. We have now added zoomed-in images of Hoechst- labeled single

© 2023. Published by The Company of Biologists under the terms of the Creative Commons Attribution License (https://creativecommons.org/licenses/by/4.0/).

spermatocytes in control and *VCP*-RNAi testes with phase contrast images to view the nucleolus (Fig. 2D). These images show that chromosomes do in fact move to distinct territories in *VCP*-RNAi spermatocytes (lines 168-170), and the chromatin actually appears to be hyper-condensed (lines 177-179). Notably, we did not observe a noticeable difference between nucleoli in control and *VCP*-RNAi spermatocytes (lines 177-179). The X-chromosome is also visible near the nucleolus and brightly labeled by Hoechst staining, suggesting that it is indeed condensed.

We have also now added data suggesting that the X-chromosome is properly inactivated and that the Y-chromosome is active. First, we used an antibody against phospho-serine 2 RNA Polymerase II (Pol II S2p), which a recent study used to detect transcriptional activity at the X-chromosome (Mahadevaraju et al., 2021. *Nat Comm*). In both control and *VCP*-RNAi spermatocytes, the X-chromosome was not labeled by Pol II S2p, but autosomes were (Fig. 3C; lines 221-224), suggesting that the X-chromosome is inactivated. We also performed RT-qPCR to measure the expression of a Y- chromosome gene, *kl-3*. We found that expression of *kl-3* exon 2, which is expressed early in the spermatocyte stage (Fingerhut et al., 2019. *Plos Genet*), was not significantly affected by knockdown of *VCP* (Fig. 2G; lines 189-196), suggesting that the Y-chromosome is active. In contrast, the expression of the more distal *kl-3* exons 6 and 14, which are only expressed later, was significantly reduced when *VCP* was knocked down (Fig. 2G; lines 189-196), which is consistent with a developmental arrest early in the spermatocyte stage.

Collectively, these added data demonstrate that VCP is required for progression beyond early spermatocyte stages and for proper regulation of nuclear size, but is dispensable for the formation of chromatin territories, nucleolar morphology, and sex chromosome dynamics in spermatocytes.

c.The H2Aub staining does not appear to fully overlap DAPI in the GCs but it does appear to fully overlap in somatic cyst cells, but it's hard to tell at the current resolution. A zoomed in view of single nuclei would be nice. DAPI dark versus DAPI light? Territories?

The reviewer's observation that H2Aub does not completely overlap with Hoechst labeling, as it does in cyst cells, is correct. Instead, H2Aub signal appears uniform throughout the spermatocyte nucleoplasm in *VCP*-RNAi testes. To more clearly show H2Aub labeling in spermatocyte nuclei, we have added insets of stage-matched spermatocytes to the images in Fig. 4A. Single-channel images are shown in grayscale. Hoechst is on the far left, H2Aub in the middle, and merge (Hoechst, blue; H2Aub, magenta) on the far right. This is an interesting phenotype given that chromosomal territories are apparent. The presence of bright H2Aub signal in the nuclear interior may be indicative of H2Aub that has been removed from chromatin, but cannot be extracted from spermatocyte nuclei in the absence of VCP. We have included a statement on this result in lines 256-259.

2)The authors should cite and discuss some of the relevant single cell data as it pertains to the sharp expression transition e.g. PMID: 33563972 PMID: 34403408 PMID: 35239393

Thank you for this suggestion. We have now cited these articles when we state that "extensive transcriptional rewiring occurs at meiotic prophase" (lines 66-68). We have also cited and discussed the articles that specifically addressed sex chromosome activation status in spermatocytes when describing experiments to test sex chromosome activation status in *VCP*-RNAi testes (lines 218-224).

3)"Available on request" does not hack it anymore. Please have the authors deposit all relevant data in a repository. These exist for virtually all data types. See Figshare, Open Science, Zenodo, etc. Development may have preferences.

Thank you for pointing this out. We have now deposited all relevant data to Dryad, as recommended by Development. See the link below.
https://datadryad.org/stash/share/nSOkSOUxJFYsl1quL3vRnK9SlofPLpVxACPFonFGt_Q

4)Thank the authors for using stock center ids. Using shorthand for alleles and lines creates confusion in the literature and overtaxes already overextended FlyBase staff. Please take one more step and use FlyBase identifiers and preferably use the FlyBase Alleles and Reagent Table (ART).
https://wiki.flybase.org/wiki/FlyBase:Author_Reagent_Table_(ART).

© 2023. Published by The Company of Biologists under the terms of the Creative Commons Attribution License (https://creativecommons.org/licenses/by/4.0/).

Yes, we agree that this is good practice. We have now included FlyBase strain identifiers for each commercially available strain used in this study (see methods; lines 520-532).

Minor

5)It seems to me that VCP and tTAFs are required for the transcriptional program. I'd play with the title a bit, along the lines of Figure 7.

We have now updated our title to "VCP promotes tTAF-target gene expression and spermatocyte differentiation by downregulating mono-ubiquitinated H2A". We believe that this encapsulates all of the main findings presented in our paper.

6)110: "stem cells, spermatogonia, and spermatocytes" or the like, would be better than "mostly spermatocytes".

Thank you for the suggestion. We agree that this specific wording could be improved. We have now updated this part of the text to "stem cells, spermatogonia, and spermatocytes, but not later germ-cell stages" (lines 114-117) to more accurately describe the composition of larval testes.

7)The Authors could be a bit bolder is some areas, for example, state explicitly what rescue of VCP by H2Aub KD means. Readers are busy, you have to hit them over the head when you have a critical result to explain.

We agree, and we have updated the text to be more explicit. For example, when summarizing the results from our double knockdown gene expression experiments (Fig 6), we now state "Collectively, these data indicate that VCP downregulates H2Aub to drive the expression of several tTAF-regulated genes." See lines 363-364. Similarly, when summarizing the results from our developmental analyses in Fig. 7, we now state "Thus, while it appears that VCP must also execute additional functions to support germline development past the elongating spermatid stage, we concluded that VCP downregulates H2Aub in spermatocytes to promote spermatocyte differentiation." See lines 439-442. We believe that these statements are more explicit and more clearly convey the significance of these experiments.

8)I enjoyed most of the supplemental figures and think that the authors might be able to incorporate some of their favorites into the smallish main figures. If they want to make room, the later stages of spermatogenesis might not be needed in the main figures, given the spermatocyte block.

Thank you for the suggestions. This was a point also raised by Reviewer 2. As part of the revisions, we have now added significant new data, and the main figures are more substantial than in the original submission. Our manuscript now includes seven main data figures (plus one model/schematic). In all cases, we have made every effort to maximize inclusion of the most important data in the main figures (up to size restrictions specified by *Development*) and to limit the size and amount of supplementary data. In the revised submission, we present an equal number of supplementary data figures as main data figures (i.e., seven each), and the supplementary data figures are generally smaller in content; perhaps two exceptions are S6 and S7, but we cannot make the corresponding main figures any larger based on size restrictions. We have now combined data from what used to be Fig. S6 and Fig. S7 together. We have also moved some data from the supplement to main figures. For example, we moved images showing that double knockdown of *VCP* and *sce* blocks H2Aub in spermatocytes from what used to be Fig. S6A to what is now Fig. 6A. While the supplemental figures do provide important information that adds robustness to our study, we believe that all data that are essential to understand the major findings of our study are in the main figures.

9)The particular drivers and antibodies have been well described, but it is always nice to state that the patterns match expectations. It never hurts to do at least a few quick experiments with additional drivers.

This a valid point. We have now explicitly stated that the knockdown of *VCP* with BamGal4 specifically-knocked down *VCP* expression in spermatocytes, but did not affect *VCP* expression in

© 2023. Published by The Company of Biologists under the terms of the Creative Commons Attribution License (https://creativecommons.org/licenses/by/4.0/).

spermatogonia and cyst cells, and thus matched our expectations (lines 150-153). These data are shown in Fig. S2A. Additionally, we now state that knockdown of *sce* with BamGal4 also matched our expectations, as H2Aub is still present in spermatogonia and cyst cells (Fig. 6A; lines 349-351).
We also used VasaGal4 to drive the expression of an ATPase-dead allele of *VCP* (*VCP*^{K2A}), and found that H2Aub was not downregulated in spermatocytes (Fig. 4D; lines 275-278).
Therefore, we believe the drivers used in our study are the most suitable for the purposes of this study, and the addition of other drivers is superfluous to the study at hand.

Reviewer 2 Advance Summary and Potential Significance to Field:
In their manuscript, Butsch and coauthors investigate the role of Valosin- containing protein (VCP) in the Drosophila male germline. They demonstrate that VCP is cytoplasmic in mitotic spermatogonia and translocates to the nucleus in meiotic spermatocytes. In turn nuclear translocation of VCP in primary spermatocytes is dependent on testis-specific TBP-associated factors (tTAF) and is required to drive spermatocyte differentiation. Indeed VCP-RNAi testes do not contain germ cells that develop beyond the spermatocyte stage. They further show that nuclear VCP is required for downregulating a repressive histone modification, mono-ubiquitinated H2A (H2Aub), leading to spermatocyte differentiation.

Remarkably, inhibiting PRC1 ubiquitin ligase activity in the absence of VCP, is sufficient to overcome the meiotic-arrest phenotype and to promote development through meiosis, indicating that H2Aub downregulation is an important function of VCP in spermatocyte differentiation.
This manuscript provides a significant contribution to our understanding of the mechanisms underlying Drosophila spermatogenesis. Thus, this paper is suitable for publication in Development.

Reviewer 2 Comments for the Author:
I suggest minor issues that the authors need to address before the manuscript can be accepted.
1)The sentence in the abstract (lines 30-32): “Like tTAF mutants, spermatocyte gene expression fails to properly activate in VCP-RNAi testes, and germ cells arrest in early meiosis” is not clear and should be rephrased.

Thank you for pointing this out. Upon re-reading this sentence, we can now see how this sentence could be confusing. To make this sentence clearer, we have updated it to now read “VCP promotes the expression of several tTAF-target genes, and *VCP* knockdown, like tTAF loss-of-function, causes cells to arrest in early meiotic stages.” See lines 29-31. We believe that this sentence now clearly and more directly states what VCP does in spermatocytes, while also connecting VCP to tTAFs.

2)There are too many supplemental figures. On the other hand, some figures contain few panels. I think that the authors can reorganize the figures to show essential information in the main figures.

Thank you for pointing this out. This was a point also raised by Reviewer 1.
As part of the revisions, we have now added significant new data, and the main figures are more substantial than in the original submission. Our manuscript now includes seven main data figures (plus one model/schematic). In all cases, we have made every effort to maximize inclusion of the most important data in the main figures (up to size restrictions specified by *Development*) and to limit the size and amount of supplementary data. In the revised submission, we present an equal number of supplemental figures as main figures (i.e. seven each), and the supplemental figures are generally smaller in content; perhaps two exceptions are S6 and S7, but we cannot make the corresponding main figures any larger based on size restrictions. We have now combined data from what used to be Fig. S6 and Fig. S7 together. We have also moved some data from the supplement to main figures. For example, we moved images showing that double knockdown of *VCP* and *sce* blocks H2Aub in spermatocytes from what used to be Fig. S6A to what is now Fig. 6A. While the supplemental figures do provide important information that adds robustness to our study, we believe that all data that are essential to understand the major findings of our study are in the main figures.

3) The panels Figure 4 and Figure S5 show western blots of poor quality. Overloading of loading control (actin) makes it difficult to quantify the difference between the mutant and the control.

We apologize for this. We had unintentionally overexposed the actin panels in the previous blots by

© 2023. Published by The Company of Biologists under the terms of the Creative Commons Attribution License (https://creativecommons.org/licenses/by/4.0/).

increasing image brightness post-acquisition, thus making the lanes appear overloaded. We are now including different representative blots where the Actin control is appreciably not overloaded. All quantifications were done on raw blot images in which brightness and contrast were not adjusted.

5)The authors say: “Combined knockdown of VCP and sce allowed germ cells to develop beyond the spermatocyte stage to the round spermatid stage, indicated by compact chromatin morphology and accumulation of mitochondria in a nebenkern structure (Fabian and Brill, 2012) adjacent to germ-cell nuclei (Fig. 6D).” It is weird to show the nebenkern by using mitotracker staining of mitochondria. Could it be possible to show phase contrast images of spermatids in the three genotypes?

We have now used phase contrast microscopy to show spermatids in control (BamGal4/+) and DKD (BamGal4>*VCP*-RNAi, *sce*-RNAi) testes. *VCP*-RNAi testes do not form spermatids, and hence images of spermatids cannot be shown in this genetic background. In the control images (Fig. 7C), we show elongating spermatids, indicated by the protein body and elongated mitochondria. In the DKD images (Fig. 7C), we show cells that have features of round and elongating spermatids; a protein body is present at DKD spermatid nuclei, but mitochondria are circular and fail to elongate. Interestingly, although we observed a nebenkern via fluorescence microscopy of MitoTracker labeling, we did not observe a phase-dark nebenkern by phase contrast, suggesting potential spermatid mitochondrial defects in the absence of VCP. We discuss these data in lines 391-400. We have also now included phase contrast images of elongating spermatids in *VCP*-RNAi sce^{KO} MARCM testes (Fig. 7E), and these are compared to spermatocytes in *VCP*-RNAi sce^{WT} MARCM testes (Fig. 7E). Similar to DKD testes, we observed a protein body at spermatid nuclei in *VCP*-RNAi sce^{KO} MARCM testes (Fig. 7E). We have discussed these data in lines 427-436. We have also added phase contrast images of spermatocytes in control and *VCP*-RNAi testes to Fig. 2C, D.

Overall, phase contrast imaging throughout our study has provided important information regarding cell stage, which we could not have obtained using the imaging methods in our initial submission. Thank you for this suggestion.

***** Reviewer 3 Advance Summary and Potential Significance to Field:
Overall the paper provides and interesting advance for the field. The finding that VCP translocation to the nucleus is important for down regulation of H2Aub, for ensuring normal spermatocyte development, and for ensuring normal spermatocyte transcription are all well supported and point to a previously unknown aspect of regulation in these cells. There are some issues with data presentation, and some areas where the logic of the experimental design was not clear to me.

Reviewer 3 Comments for the Author:
Introduction: As a spermatogenesis expert rather than a VCP expert, I would like just a little more information in the introduction about what this protein does molecularly, as written it is clear it is in lots of cellular processes, but not how it works. What happens to ubiquitinated cargo after VCP has bound it.

Thank you for bringing this to our attention. In the first sentence of the Introduction, we now provide more details regarding the molecular function of VCP (lines 39-41). We have also provided additional information stating that VCP functions as a hexamer when introducing the VCP^{K2A} allele and describing why the overexpression of this allele generates dominant-negative effects (lines 266-269).

Results:
Figure 1, localisation change to nucleus in primary spermatocytes is very clear. It is also clear from the pictures that it is excluded from the nucleolus (and maybe enriched on Y- loops?). The exclusion from nucleolus is relevant to the context of PRC1 and tTAFS, since both of them are enriched in the nucleolus at this stage. This should be mentioned and commented on.

These are good points. We have now added more data to provide a more detailed characterization of VCP localization, which we describe in detail below.

© 2023. Published by The Company of Biologists under the terms of the Creative Commons Attribution License (https://creativecommons.org/licenses/by/4.0/).

To clearly show that VCP is excluded from the nucleolus in spermatocytes, we labeled nucleoli using an antibody against a nucleolar protein, Fibrillarin, in VCP-GFP testes. In these images, VCP-GFP signal does not co-localize with Fibrillarin (Fig. 1F; lines 134-135), indicating that VCP is indeed excluded from spermatocyte nucleoli.

Very nice observation that VCP is enriched at Y-loops. We investigated this possibility further by imaging VCP-GFP spermatocytes at high magnification and examining the subnuclear localization of VCP. Indeed, VCP-GFP signal was brighter in the interior of the nucleus (Fig. 1G; lines 135-141), where Y-loops are located (Bonaccorsi et al., 1988, *Genetics*; Cenci et al., 1994, *J Cell Sci*; Mahadevaraju et al., 2021, *Nat Comm*) and actively transcribed (Fingerhut et al., 2019, *Plos Genet*). We confirmed that VCP is enriched in the interior of spermatocyte nuclei relative to peripheral autosomes by quantifying VCP-GFP intensity at Hoechst-positive autosomes (outlined in Fig. 1G) and the Hoechst-negative interior. VCP-GFP intensity was significantly higher in the Hoechst-negative interior than at Hoechst-positive autosomes (Fig. 1H; lines 135-141). This quantification indicates that VCP is indeed enriched in a similar location as Y- loops.

This subnuclear localization pattern of VCP is notable, because it does not overlap with tTAFs and Sce, which are primarily localized in nucleoli (Chen et al., 2005, *Science*), or with Polycomb (Pc), which localizes to autosomes and nucleoli (Chen et al., 2005, *Science*). Collectively these observations reinforce our argument that tTAFs likely do not directly interact with VCP to support nuclear entry of VCP (lines 457-461). However, these findings do not necessarily indicate that VCP would not directly interact with PRC1 components. For example, a VCP-PRC1 interaction could be transient. In theory, VCP may serve as a barrier to PRC1 components from entering the interior of the nucleus (lines 497-504). In the future it will be interesting to investigate whether VCP regulates PRC1 localization and activity.

For figure 1 (and later figures) it's really hard to see the DNA when shown in blue. And the single channel images should be presented in grayscale throughout. (https://www.youtube.com/watch?v=JT9mUkEG-C0) explains why.

Thank you for this helpful suggestion. We have now updated our images to show all single channel DNA images in grayscale. More generally, we have updated the other single channel images presented in the paper to grayscale as well, with the exception of H2Aub and one VCP-GFP image. We have shown almost all H2Aub single channel images using the Fire LUT (Fiji), which we feel more strongly conveys the striking downregulation of H2Aub as cells transition from spermatogonia to spermatocytes. We have also used this same LUT when we show that VCP is enriched in the nuclear interior of spermatocytes (Fig. 1G), which was not as obvious in grayscale. Overall, we believe our images now more clearly illustrate our points and support our major conclusions.

RNAi experiments. It's worth reminding the reader that null mutants are lethal, and that the gene is essential for cell viability. Fig S2, would be nice to see the GFP only channel separated out and in grayscale. The protein remaining in spermatogonia and in cyst cells would be more obvious.

It is a good point that null VCP mutants are lethal, which was previously reported (León and McKearin, 1999, *Mol Biol Cell*). We have now included a brief explanation of why we chose to use RNAi to assess VCP function (lines 148-153). We have also updated Fig. S2 to show VCP-GFP alone in grayscale, with arrows marking cyst cells that retained VCP.

Figure 2. The RNAi testes are clearly much smaller than WT. It would be better to use phase contrast microscopy rather than DIC for this imaging. It is very hard to see the morphology of the spermatocytes in the RNAi testes. It is not clear if they are earlier or later spermatocyte stages as shown. The Hoechst labelling sort of helps, but again is very hard to interpret. The statement that the arrest is at the point that VCP enters the nucleus needs more data to support it. VCP seems to enter the nucleus in very early spermatocytes, but the precise arrest point seems to be later. The down regulation of tTAF target genes is clear, but a causal link is harder to make. The selected genes increase in expression as spermatocytes mature. If the RNAi causes an arrest early in spermatocytes these genes would be reduced in expression because the cells remain early, not because of a direct transcriptional effect. It would be good to test a gene expressed in primary spermatocytes that does not depend on tTAFs. An in situ hybridisation could also help confirm the reduction in the RNAi spermatocytes.

© 2023. Published by The Company of Biologists under the terms of the Creative Commons Attribution License (https://creativecommons.org/licenses/by/4.0/).

Thank you for these helpful points. We have decided to keep the original DIC images, as we believe they provide valuable information regarding the striking size difference between control, *VCP*-RNAi, and DKD testes (Figs. 2A, 7A), as well as germ-cell cyst degeneration (Fig. 7A, B). But, we have now also added phase contrast images of spermatocytes in control and *VCP*-RNAi squashed preparations (Fig. 2C, D). These images show spermatocytes, round spermatids, and elongated spermatid tails in a control preparation, and an accumulation of spermatocytes in *VCP*-RNAi squashes (lines 163-171). Importantly, we did not observe cells that developed beyond the spermatocyte stage in *VCP*-RNAi testes. Based on chromatin morphology, *VCP*-RNAi spermatocytes appear to arrest in the S3-S4 stage (Cenci et al., 1994. *J Cell Sci*). Additionally, expression of *kl-3* exon 2, which is expressed early in spermatocyte development (Fingerhut et al., 2019. *Plos Genet*), was not significantly affected by knockdown of *VCP*, but expression of *kl-3* exons 6 and 14, which are expressed in later stages, was significantly reduced (Fig. 2G; lines 189-196). Together, these data support the conclusion that VCP is required for progression beyond early stages of spermatocyte development, which is shortly after VCP enters the nucleus (lines 197-199).

We have now normalized spermatocyte gene expression data to *cyclin A* expression, which is not regulated by tTAFs (White-Cooper et al., 1998. *Development*) and was also not affected by knockdown of *VCP* (relative expression of 2.95 in control and 3.04 in *VCP*-RNAi). After normalizing the expression values to *cyclin A* expression, we still observe a significant decrease in spermatocyte gene expression in *VCP*-RNAi testes (Fig. 3E). Notably, we found that double knockdown of *VCP* and *sce*, which blocks H2Aub, significantly increased spermatocyte gene expression compared to *VCP* knockdown alone, even after normalizing to *cyclin A* expression. Thus, our data now confirm that VCP promotes the expression of spermatocyte genes via H2Aub downregulation.

The increase in H2Aub in RNAi spermatocytes is very clear.

I am confused by the overexpression experiments with the auxin system. If the constructs are only being driven in the germline (vasaGal4), why do you need the Gal80-AID and auxin at all? Why keep the flies on auxin for 10 days? Why not use bamGal4 for this experiment? What effect does the knock out of the ATPase activity have on the molecular function of VCP, ie why is this dominant (this links back to what exactly does VCP do)?

Surprisingly, BamGal4- and VasaGal4-driven expression of the K2A allele without the use of an inducible system is lethal, hence the need for the auxin-inducible system. We have now further described the reasoning behind this experimental approach and explained why the overexpression of VCP^{K2A} yields dominant-negative effects in lines 266-272. Briefly, VCP functions as a hexamer; thus, overexpression of an ATPase-dead allele, such as VCP^{K2A}, generates hexamers containing the wild-type and mutant proteins, which impedes function.

Screen of co-factors. “we screened several… and found one…”. It would be very nice to know which ones did not give you a phenotype too.

Thank you for indicating this, as we agree that it would be helpful to convey this information. We have now included a table listing the genes we tested and whether knockdown of these genes yielded an increase in H2Aub (Fig. S6A).

The old vs new H2A experiment is intriguing, but I wonder about technical issues. The main text should state that the H2A construct is under UAS control, thus you are only assaying protein that has been made in the spermatocytes, and that is in addition to endogenous protein. The hs-flp excision is clearly very efficient in the WT testes, as all cells are red, not green. Are you sure that flp was so efficient in the mutant? In the RNAi testes there is variability between cysts, but not within cysts, how is the H2A turnover coordinated between cells within a cyst? How does the proposed inter-cyst variability in H2A turnover rate correlate with the very uniform (between cysts) up regulation of H2Aub seen in the other stainings?

These are good questions. To determine whether our results could be caused by impaired FLP-mediated excision, we designed a forward primer against the UAS sequence and a reverse primer against mCherry to amplify the H2A-turnover transgene. Importantly, because neither of these

© 2023. Published by The Company of Biologists under the terms of the Creative Commons Attribution License (https://creativecommons.org/licenses/by/4.0/).

sequences are excised by FLP, we were able to detect the full-length transgene as well as the partial H2A-mCherry transgene made by recombination (i.e., when H2A-GFP has been excised) using the same primer set. We performed PCR on DNA extracted from heat-shocked control and *VCP*-RNAi testes and found that FLP activity was not significantly affected by the knockdown of *VCP*, based on the bright H2A-mCherry bands and dim full-length bands. A gel image of this experiment is in Figure S6B, and we discuss this experiment in lines 302-307.

The apparent inter-cyst variability of H2A turnover is an interesting phenotype. Admittedly, we do not have a definite explanation for this phenotype at this point (lines 322-323); however, it is robust and reproducible, and not just an experimental fluke. To more clearly show that this phenotype is reproducible, we have now included additional images of four control and four *VCP*-RNAi testes expressing the H2A-turnover transgene (Fig. S6C). In control testes, GFP signal is largely absent, and we observe fairly uniform, bright mCherry signal. These images are consistent with the images shown in Fig. 5B and indicate that old H2A is robustly turned over in control testes. However, in all *VCP*-RNAi testes (Fig. 5B, Fig. S6C), we observed bright GFP and dim mCherry signal in many of the cysts. Though other cysts showed dimmer relative GFP, the mean GFP intensity and the GFP/mCherry ratio for the spermatocytes in each testis were consistently higher in *VCP*-RNAi testes compared to controls (Fig. 5C, D). Importantly, when quantifying GFP and mCherry intensities, we outlined all visible spermatocytes in the fields of view, so that we measured the mean GFP and mCherry intensities in the entire imaged spermatocyte population. Therefore, our quantification accounts for the inter-cyst variability phenotype and demonstrates a general retention of more GFP signal (even relative to mCherry) upon *VCP* knockdown. These quantifications support our fundamental conclusion that VCP enables robust H2A turnover in spermatocytes (lines 322-326). Because we believe our method of scoring is important to clarify this point, we have now included a description of how we performed this quantification in the Methods (lines 687-688).

We agree that it is important to make the point that this approach allows us to monitor fluorescently tagged, transgenic H2A, but that it does not allow us to track turnover of endogenous H2A. We now explicitly state this (lines 297-298). It is possible that the turnover of endogenous H2A in cysts may be somewhat stochastic; the exact timing is impossible to tell from our approach, as we are not monitoring endogenous H2A. Moreover, the expression of the H2A-turnover transgene may be somewhat variable between cysts. With these even slight variations, we believe that some cysts may be more prone to overloading with endogenous and transgenic H2A, which could further slow H2A turnover in the already H2A turnover-impaired *VCP*-RNAi testes. Such overloading could potentially spread throughout a cyst uniformly, due to connections by ring canals. Additionally, it is also possible that *VCP* knockdown could be variable between cysts. To avoid inducing FLPase expression after the initial heat shock, we kept the flies at 25°C (as opposed to 29°C), which could lead to reduced knockdown of *VCP* in some cysts and variable H2A turnover. As we now state in the Discussion (lines 488-492), an approach to measure the turnover of endogenous H2A would likely provide further information regarding the possible intra- and inter-cyst regulation of this event and how these complexities in control may contribute to spermatocyte gene expression. While these considerations thus inspire interesting questions for future investigation, we maintain that our results conclusively indicate that H2A turnover in spermatocytes is regulated by VCP, which is the main conclusion for the study at hand (see above).

As the reviewer notes, we do observe a fairly uniform distribution of H2Aub in *VCP*- RNAi spermatocytes (Fig. 4A). However, there are two key differences between the experimental setup used in this experiment (Fig. 4A) and in the H2A-turnover experiment (Fig. 5B-D). First, flies for the H2Aub-labeling experiment were housed at 29°C for seven days prior to dissection to boost the strength of *VCP* knockdown, whereas flies for the H2A-turnover experiment were housed at 25°C after heat shock for five days. Thus, there are likely differences in RNAi efficiency between these two experiments. Second, when labeling H2Aub with the antibody, we did so in testes that did not overexpress H2A; therefore, we labeled only endogenous H2A in this case (and there was not extra, exogenous H2A around). However, in the H2A-turnover experiment, the approach required that we overexpress H2A. Due to these differences, we think it is difficult to directly compare these two experiments, though each can give us complementary, useful information (i.e., knockdown of *VCP* leads to an increase in endogenous H2A and inhibits the capacity of spermatocytes to turn over H2A produced off of a transgene).

Fig S8A, the labels above the middle and right hand panels are switched around. The data shown in

© 2023. Published by The Company of Biologists under the terms of the Creative Commons Attribution License (https://creativecommons.org/licenses/by/4.0/).

figure S8C seems quite important to the message of the paper – why is this a supplement? I agree that the VCP-RNAi, sceKO clones do make cells that look like post-meiotic stages, but these are extremely abnormal (phase contrast would be nice here too).

Thank you pointing out this labeling error. We have now ensured that our figures are properly labeled.

We agree that although identifiable spermatids form in DKD testes as well as in *VCP*- RNAi, *sce*^{KO} testes, these spermatids show some peculiarities. To investigate spermatid structure further, we have now used phase contrast microscopy. First, we compared spermatids in control (BamGal4/+) and DKD (BamGal4>*VCP*-RNAi, *sce*-RNAi) testes.
In the control images (Fig. 7C), we show elongating spermatids, indicated by the protein body and elongated mitochondria. In the DKD images (Fig. 7C), however, we show cells that have features of round and elongating spermatids; a protein body is present at DKD spermatid nuclei, but mitochondria are circular and fail to elongate. Interestingly, although we observed a nebenkern via fluorescence microscopy of MitoTracker labeling (Fig. S7A), we did not observe a phase-dark nebenkern by phase contrast (Fig. 7C), suggesting potential spermatid mitochondrial defects in the absence of VCP. We discuss these data in lines 381-395. We also performed phase contrast imaging of *VCP*-RNAi, *sce*^{KO} MARCM testes. This revealed similar themes; while we observed a protein body at spermatid nuclei in *VCP*-RNAi, *sce*^{KO} MARCM testes, we did not observe a phase-dark nebenkern (Fig. 7E). Moreover, as in DKD testes, mitochondria labeled by MitoTracker were circular and not elongating (Fig. S7E). We have discussed these data in lines 427-436. Given these multiple lines of evidence, we view the spermatids that form upon double inhibition of *VCP* and *sce* to represent a hybrid and/or transitional state somewhere between the round and elongating spermatid stages. Thus, while it appears that VCP must also execute additional functions to support germline development past this hybrid and/or transitional spermatid stage, these data strongly support the conclusion that VCP downregulates H2Aub in spermatocytes to promote spermatocyte differentiation, because we never observed post-meiotic spermatids when *VCP* was inhibited alone.

We also agree with the reviewer that these data are important to the message of the paper. We have now included the new phase contrast images for DKD testes in Fig. 7C and for MARCM testes in Fig. 7E. Several other images related to this set of experiments needed to be included in supplement (Fig. S7) due to figure-size restrictions.

I gave this manuscript to an early career researcher to comment on. Her feedback is below. (some of this overlaps with comments I have already made above)

Line 41. “ubiquitin-selective protein segregase” - I'm not sure I know what this is without more explanation.

We agree that this statement is not as clear as it could be and have now modified this statement to make it clearer. This sentence now reads “Valosin-containing protein (VCP) is a broadly expressed protein that functions as a hexameric AAA+ ATPase to regulate protein homeostasis by binding ubiquitinated substrates and targeting them for degradation.” See lines 39-41.

Line 51. Stage specific transcriptomic data… Fly Cell Atlas shows it is highest in spermatocytes, also spermatogonia in and cyst cells. This correlates with your protein data.

Yes, this is correct; we indicate this point in the results (lines 117-119).

Line 127-128 (bam-RNAi). Images look convincing. However, why do it this way? What if nuclear translocation is bam dependent? Is this really necessary if you can just count the number of spermatogonia/spermatocytes in a cyst? Surely this will tell you when translocation occurs? I would like to see some pictures of individual cysts.

Nuclear translocation of VCP is absolutely Bam-dependent; we show this in Fig. S1. This is not surprising, because Bam is required for the spermatogonia to spermatocyte transition (McKearin and Spradling, 1990, *Genes Dev*) and we do not observe VCP in spermatogonia nuclei (Fig. 1C). However, this dependency is almost certainly indirect. We show that VCP depends on tTAFs to enter

© 2023. Published by The Company of Biologists under the terms of the Creative Commons Attribution License (https://creativecommons.org/licenses/by/4.0/).

spermatocyte nuclei (Fig. 3A, B), which are not expressed until after Bam is already gone; thus, it seems unlikely that Bam would directly influence VCP localization. Rather, some factor under control of tTAFs seems more likely to directly regulate VCP localization in spermatocytes, as we note in the Discussion (lines 462-466).

In regard to co-labeling with a cyst marker: We think the timing of nuclear translocation is relatively clear with the data presented. By co-labeling with Hoechst (DNA), we observed that VCP is seen in the germ-cell nuclei that show tri-lobed chromatin (spermatocytes), but not in immediately preceding germ cells with compact chromatin (spermatogonia). To make this point clearer, we have now added an individual Hoechst/DNA panel to Fig. 1C. Again, as noted above, these data fit with the *bam*-RNAi results.

Line 142-144. I know the whole testis images are meant to be at the same magnification, but a larger magnification image of the VCP-RNAi testes would be really nice. It would make the meiotic arrest phenotype easier to see.

This is a good point, and a point made by other reviewers. To address this concern, we have now added phase contrast images of testis squashes (Fig. 2C; lines 163-171). In control squashes, spermatocytes, round spermatids, and elongated spermatids were all present. However, the most developed germ cells we observed in *VCP*-RNAi squashes were spermatocytes. Throughout all experiments with *VCP*-RNAi testes, we never observed post-meiotic germ cells.

Line 146-148. I don't find Fig 2C particularly clear. Perhaps more labels would help? Also images of cysts would make stage clearer as well.

We acknowledge that the original images in Fig. 2C could be improved. We have now added images of single control and *VCP*-RNAi spermatocytes with Hoechst-labeling (Fig. 2D). These images clearly show spermatocytes with well-formed nucleoli and fairly normal chromatin morphology. Based on chromatin morphology and the fact that Y- chromosome gene regions, which are not expressed until late spermatocyte stages, are significantly downregulated in *VCP*-RNAi testes (Fig. 2G; lines 189-196), we conclude that cells arrest early in spermatocyte development.

Page 153-155. Could test this by making a mutant for which the protein doesn't localise in the nucleus, but probably not for this paper.

This is true, but, as noted by the Reviewer, this is beyond the scope of this particular study. As we note in the Discussion (lines 462-466), the mechanism controlling VCP nuclear entry in this developmental context is currently not well understood; thus, it is currently not feasible to make a *VCP* mutant that would be specifically defective in this regulatory step. However, we acknowledge that this is a logical next step in investigating this topic, and we now explicitly highlight this as an important task for the future (lines 466-468). We maintain that our findings indicating that H2Aub, which is only present in the nucleus, is downregulated by VCP (Fig. 4) to drive gene expression (Fig. 6D) and meiotic progression (Fig. 7C, E) strongly suggest that VCP does indeed play an important role in spermatocyte nuclei.

Page 158-160. Why test tTAFs and not also tMAC?

We focused on tTAFs because they have previously been studied in the context of PRC1 (Chen et al., 2005. *Science*; Chen et al., 2011. *Development*). Additionally, spermatocyte nucleoli form normally in tTAF mutants (Lin et al., 1996. *Development*), similar to what we see in *VCP*-RNAi spermatocytes (Fig. 2C, D). However, nucleoli are oddly shaped in tMAC mutants (Lin et al., 1996. *Development*). Lastly, cells actually skip meiotic divisions and transition from the spermatocyte stage to the round spermatid stage in tMAC mutants (Lin et al., 1996. *Development*), which we did not observe in *VCP*-RNAi testes. Collectively, these contrasting phenotypes between tMAC mutants and *VCP*-RNAi testes suggest that VCP does not function in the same pathway as tMAC.

Line 242-244. I got a bit lost here

This statement indicates that the proteasome is important for VCP to enter spermatocyte nuclei, which impedes our ability to determine whether the proteasome is also directly involved in the

© 2023. Published by The Company of Biologists under the terms of the Creative Commons Attribution License (https://creativecommons.org/licenses/by/4.0/).

downregulation of H2Aub. It could be that *rpt2*-RNAi indirectly leads to H2Aub accumulation simply because this approach prevents VCP from going into the nucleus in the first place. Thus, it is hard to distinguish whether H2Aub is accumulating because the proteasome is not degrading it or because VCP is not in the nucleus. We have now slightly adjusted this statement to more clearly convey this point (lines 334-338). It would be ideal if we could force VCP into the nucleus when the proteasome is inhibited to determine whether the proteasome does indeed degrade H2Aub, but, with our current state of knowledge, such an experiment is not feasible.

Line 245-256. Having now read this section, I feel like this should be 'partially suppresses' or something like that. I guess the DKD aren't technically meiotic arrest phenotype anymore... but they haven't been fully rescued.

Because cells in DKD testes no longer arrest at the spermatocyte stage, we believe stating that genetically inhibiting H2Aub promotes spermatocyte differentiation and meiotic progression is accurate. We do not more broadly state that inhibiting H2Aub in the absence of VCP rescues sperm development, which would require such a qualification. Thus, we believe our wording is appropriate.

Line 303-304. I could do with a diagram.

Thank you for the suggestion. We agree that this experimental setup can be confusing. To clear up some of this confusion, we have now included a diagram illustrating the genotype of GFP-positive and GFP-negative cells in *VCP*-RNAi, sce^{WT} and *VCP*-RNAi, sce^{KO} testes to the main text (Fig. 7D).

Line 313-314 So what stage do the DKD testes get to? They mention onion stage, but any further? Any elongation at all? (Histone ubiquitination in canoe stage nuclei, do VCP-RNAi get to this stage?)

We apologize for the confusion; please see our response above to a similar question. To gain more detailed information regarding spermatid structure and staging in DKD testes, we have now used phase contrast microscopy to visualize spermatids in control (BamGal4/+) and DKD (BamGal4>*VCP*-RNAi, *sce*-RNAi) testes. In the control images (Fig. 7C), we show elongating spermatids, indicated by the protein body and elongated mitochondria. In the DKD images (Fig. 7C), we show cells that have features of round and elongating spermatids; a protein body is present at DKD spermatid nuclei, but mitochondria are circular and fail to elongate. Interestingly, although we observed a nebenkern via fluorescence microscopy of MitoTracker labeling, we did not observe a phase-dark nebenkern by phase contrast, suggesting potential spermatid mitochondrial defects in the absence of VCP. Thus, our data indicate that cells reach the spermatid stage in DKD testes, but based on mitochondrial morphology, they do not appear to initiate elongation. We discuss these data in lines 388-400.

Line 423-425. Maybe I've missed it but I can't find what tool they used to measure relative fluorescence intensities for all of those comparisons between cytoplasmic and nuclear VCP-GFP in their various RNAi lines.

Thank you for pointing this out. We have now added a section in the methods that describes how VCP-GFP quantification was performed (lines 577-589). Briefly, we used an image processing software, Fiji, to measure VCP-GFP intensity in nuclear and cytosolic regions of spermatocytes and spermatogonia.

Line 761-763 I'm unclear whether the test was between normal and each RNAi line, or whether the two RNAi lines were also compared.

We only compared relative expression between the RNAi line and control (i.e. control vs. *sa*-RNAi and control vs. *VCP*-RNAi). We have now added a sentence to the figure legend of Figure 3 to make this clear.

Line 786. partially rescues?

See response given above for Lines 245-256 of the original submission. In general, we are not stating that there is a full rescue to sperm development. Rather, we are stating that VCP-mediated

© 2023. Published by The Company of Biologists under the terms of the Creative Commons Attribution License (https://creativecommons.org/licenses/by/4.0/).

downregulation of H2Aub rescues development through meiosis (lines 1027-1028), which is supported by our data (Fig. 7C-E; S7A, E).

Figure 3C. Is there a sig. dif. between sa-RNAi and VCP-RNAi? If so is this because of differences in RNAi efficiency or because other stuff is involved in expression of these genes downstream of the tTAFs?

We did observe a significant difference in the expression of *janB* between *VCP*-RNAi and *sa*-RNAi testes (p = 0.0360), but not the other genes that we tested. However, we have opted not to add markings to denote statistical significance between these two groups, as this interpretation could be complicated by approach (i.e., through RNAi efficiency, as the Reviewer notes) and our intention is to emphasize that both VCP and tTAFs are needed for the normal expression of the tested genes.

Second decision letter

MS ID#: DEVELOP/2022/201557

MS TITLE: VCP promotes tTAF-target gene expression and spermatocyte differentiation by downregulating mono-ubiquitinated H2A

AUTHORS: Tyler Butsch, Olga Dubuisson, Alyssa E Johnson, and K Adam Bohnert
ARTICLE TYPE: Research Article

I am happy to tell you that your manuscript has been accepted for publication in Development, pending our standard ethics checks.

Reviewer 1

Advance summary and potential significance to field

An important addition to the literature on the transition from mitosis to meiosis in the male germline.

Comments for the author

The authors have been responsive to the reviews and should be congratulated on an excellent work.

Reviewer 3

Advance summary and potential significance to field

This paper describes a role for VCP in spermatogenesis. Relocalisation of this protein from the cytoplasm to the nucleus occurs in spermatocytes, and depends on normal spermatocyte transcriptional regulators (tTAFs). VCP depleted cells arrest, and fail to fully activate tTAF target genes. The function of VCP is mechanistically linked to H2Aub downregulation in spermatocytes. The finding that VCP translocation to the nucleus is important for down regulation of H2Aub, for ensuring normal spermatocyte development, and for ensuring normal spermatocyte transcription are all well supported and point to a previously unknown aspect of regulation in these cellss

Comments for the author

The authors have done a good job in modifying the manuscript in response to the reviews. The figure presentation is now much clearer, with a combination of grayscale and the FireLUT ensuring

© 2023. Published by The Company of Biologists under the terms of the Creative Commons Attribution License (https://creativecommons.org/licenses/by/4.0/).

that the key messages in the imaging are readily extracted. The additional experiments have added robustness to the conclusions presented, and have been appropriately interpreted. Changes to the text have been included that add clarity to the message of the paper. I have no suggestions for further revisions.

© 2023. Published by The Company of Biologists under the terms of the Creative Commons Attribution License (https://creativecommons.org/licenses/by/4.0/).
